# Machine learning and multi‐omic analysis reveal contrasting recombination landscape of A and C subgenomes of winter oilseed rape

**DOI:** 10.1002/tpg2.70209

**Published:** 2026-03-19

**Authors:** Jose A. Montero‐Tena, Silvia F. Zanini, Gözde Yildiz, Tobias Kox, Amine Abbadi, Rod J. Snowdon, Agnieszka A. Golicz

**Affiliations:** ^1^ Department of Agrobioinformatics, IFZ Research Center for Biosystems, Land Use and Nutrition Justus‐Liebig‐University Giessen Giessen Germany; ^2^ NPZ Innovation GmbH Holtsee Germany; ^3^ Department of Plant Breeding, IFZ Research Center for Biosystems, Land Use and Nutrition Justus‐Liebig‐University Giessen Giessen Germany

## Abstract

Meiotic recombination is essential for generating genetic diversity, driving plant evolution, and enabling crop improvement, yet its uneven distribution across genomes constrains breeding efforts. Here, we investigated the multi‐omic landmarks that shape the recombination landscape in *Brassica napus* by integrating epigenomic, genomic, and transcriptomic data with recombination maps derived from large multiparental rapeseed populations. Predictive machine learning accurately predicted recombination rates and hotspot location using only feature information. Recombination was generally suppressed in centromeres and other repeat‐rich, methylated regions and enriched in gene‐dense, transcriptionally active domains. Proxies for chromatin configuration—such as DNA methylation, transposable elements, or genes—consistently achieved the highest predictive power with the random forest algorithm. We discovered distinct recombination landscape patterns between subgenomes, with crossovers clustering near subtelomeres in the A subgenome and more evenly spread across the C subgenome. Models trained on A subgenome data outperformed those based on the C subgenome, although combining both subgenomes improved overall accuracy.

## Core Ideas

- We generated recombination maps in *Brassica napus* from a large set of crossovers detected across 5000 meiotic events from two large multiparental populations genotyped with a 15K single‐nucleotide polymorphism chip.
- Regarding methylation in the CHH (where H represents any nucleotide except guanine) context, we observed positive associations with recombination when restricting the analysis to transposable element (TE) bodies, reflecting the activity of CHH island silencing TEs near genes.
- We applied a machine learning approach on integrated multi‐omics and recombination data, achieving high accuracy in predicting recombination rate (overall $R^{2}$ = 0.477) and hotspot location (overall area under the receiver operating characteristic curve = 0.823). Random forest emerged as the most robust algorithm for estimating feature importance amid multicollinearity.
- We confirmed the association of well‐known chromatin state indicators—mainly DNA methylation, TEs, genes, and gene expression—in shaping recombination in *B. napus*.
- We revealed distinct recombination landscapes between the A and C subgenomes: recombination was concentrated in the subtelomeric regions of the A subgenome, whereas it was more evenly distributed along the arms of the C subgenome. These patterns aligned with differences in the distribution of chromatin state markers between the two subgenomes.

## Plain Language Summary

Meiotic recombination creates genetic diversity, which is key for plant evolution and crop breeding, but it does not occur evenly across the genome. We studied this process in rapeseed (*Brassica napus*) by combining genetic maps with data on DNA, chromatin, and gene activity. Using machine learning, we could accurately predict where recombination happens. It was rare in repeat‐rich, methylated regions like centromeres but common in active, gene‐rich areas. We also found differences between the two subgenomes: crossovers clustered near chromosome ends in the A subgenome, while they were more evenly distributed in the C subgenome.

**Abbreviations**
ALE: accumulated local effect
ATAC‐seq: assay for transposase‐accessible chromatin using sequencing
AUROC: area under the receiver operating characteristic curve
BT: boosted trees
CO: crossover
CpG: cytosine–phosphate–guanine
DT: decision tree
GC content: guanine–cytosine content
HE: homoeologous exchange
LR: linear/logistic regression
LTR: long terminal repeat
OCR: open chromatin region
RF: random forest
RNA: ribonucleic acid
SD: standard deviation
SNP: single‐nucleotide polymorphism
TE: transposable element
TPM: transcript per million
WGBS: whole‐genome bisulfite sequencing

## 1 INTRODUCTION

Meiotic recombination is the exchange of DNA between homologous chromosomes. This highly conserved mechanism is observed universally across eukaryotes (Bernstein & Bernstein, 2010) and ensures accurate segregation of chromosomes during meiosis, maintaining genomic integrity (Lambing et al., 2017). In addition, meiotic recombination promotes genetic diversity by generating new allele combinations, playing a central role in plant evolution and crop improvement (Wijnker & Jong, 2008).

*Brassica napus* (oilseed rape; rapeseed) is one of the youngest cultivated crop species and one of the most important oilseed crops worldwide. It is an allopolyploid species (AACC, 2*n* = 38) that originated from the interspecific hybridization of *Brassica rapa* (A subgenome) and *Brassica oleracea* (C subgenome), which caused a genetic bottleneck (Allender & King, 2010). Genetic diversity was further eroded by strong selection, including extreme selection for essential seed quality traits, such as oil yield or oil quality (Qian et al., 2014). To generate new genetic diversity, it is essential to harness the potential of meiotic recombination, requiring a deeper understanding of the factors underlying the frequency and positioning of meiotic recombination events in rapeseed.

Across plant genomes, recombination is not uniformly distributed. A near‐universal feature is the suppression of crossovers (COs) in centromeric regions, where recombination is constrained to ensure proper chromosome segregation (Vincenten et al., 2015). Instead, COs tend to cluster in recombination “hotspots” (Lambing et al., 2017); rapeseed exemplifies this pattern, with COs occurring in only 38% of its genome (Boideau et al., 2022). In maize, recombination is largely absent from pericentromeric regions but increases toward subtelomeric areas before decreasing at telomeric ends (Rodgers‐Melnick et al., 2015). However, this spatial pattern is not conserved across all plant species; in *Arabidopsis* and rice, for instance, recombination is distributed more broadly across non‐centromeric regions (Salomé et al., 2012; Si et al., 2015). Across plant genomes, recombination is suppressed in heavily methylated, rich in transposable elements (TEs), heterochromatic regions but favored in low‐methylation, TE‐poor, euchromatic regions with open chromatin, highlighting a conserved inverse relationship between CO frequency and both DNA methylation and TE density (Rodgers‐Melnick et al., 2015; Wijnker et al., 2013). In rapeseed, the same antagonism is seen in the distal ends, where large nonrecombinant “islands” colocalize with elevated DNA methylation (Boideau et al., 2022). These islands reinforce the idea that methylation‐mediated silencing of mutagenic TEs is a well‐conserved, principal force suppressing COs throughout the genome (Yelina et al., 2015).

Not all methylation contexts affect recombination equally. Whereas symmetrical, heritable (Law & Jacobsen, 2010) CpG (cytosine–phosphate–guanine) and CHG (where H represents any nucleotide except guanine and G represents guanine) methylation is typically anti‐recombinogenic, asymmetric CHH (where H represents any nucleotide except guanine) methylation shows a positive—albeit variable—association with CO frequency in rice (Peñuela et al., 2022), tomato, sorghum, *Arabidopsis* (Peñuela et al., 2024), and maize (Peñuela et al., 2022; Rodgers‐Melnick et al., 2015). This positive link may reflect its role in suppressing mutagenic TEs near genes, as reported in maize (Gent et al., 2013). Although genes have frequently been associated with elevated recombination rates, their predictive value appears to vary among plant species. In maize and tomato, CO regions of 10 kbp were enriched in gene‐associated features, whereas in rice and *Arabidopsis*, such regions contained fewer genes (Demirci et al., 2018). Low‐recombination regions are also associated with higher genetic load, likely due to reduced efficiency of selective sweeps and limited removal of deleterious mutations (Rodgers‐Melnick et al., 2015).

Nucleotide composition has also been implicated in shaping recombination landscapes. In maize, GC content (guanine–cytosine content) shows a strong positive correlation with CO frequency, possibly due to GC‐biased gene conversion (Rodgers‐Melnick et al., 2015). Conversely, in *Arabidopsis*, CO hotspots tend to be GC‐poor and are enriched for AT/TA dinucleotides—associated with gene promotors and open chromatin states—which also serve as predictors of CO regions in tomato (Demirci et al., 2018; Wijnker et al., 2013).

In rapeseed, the A and C subgenomes may differ in CO activity. The A subgenome exhibits a higher mean CO frequency than the C subgenome (Yan et al., 2023), in parallel with its enrichment for active chromatin marks that promote CO formation (Q. Zhang et al., 2021). Whether the genome‐wide recombination pattern in rapeseed is diploid‐like, uniform across subgenomes, or subgenome‐specific remains unresolved.

In this study, we aim to characterize the genome‐wide recombination landscape of rapeseed. We built recombination maps from detected meiotic recombination events based on single‐nucleotide polymorphism (SNP) data from large multiparental populations of German winter oilseed rape. We integrated recombination data with multi‐omics data consisting of various features associated with recombination—including DNA methylation, annotated sequences, or telomeric proximity. The power of these features for predicting recombination, as well as the interactions between them, was quantified using a machine learning approach. We confirmed some of the well‐conserved trends established previously that shape recombination genome‐wide, explored the contrast in pro‐ and anti‐recombinatory features between the A and C subgenomes, and dissected the particularities of TE body CHH methylation in rapeseed. Interestingly, we identified differences in the recombination landscape between the A and C subgenomes, with COs occurring predominantly in subtelomeric regions of the A subgenome, whereas they are more frequent in pericentromeric regions of the C subgenome.

**Core Ideas**

- We generated recombination maps in *Brassica napus* from a large set of crossovers detected across 5000 meiotic events from two large multiparental populations genotyped with a 15K single‐nucleotide polymorphism chip.
- Regarding methylation in the CHH (where H represents any nucleotide except guanine) context, we observed positive associations with recombination when restricting the analysis to transposable element (TE) bodies, reflecting the activity of CHH island silencing TEs near genes.
- We applied a machine learning approach on integrated multi‐omics and recombination data, achieving high accuracy in predicting recombination rate (overall $R^{2}$ = 0.477) and hotspot location (overall area under the receiver operating characteristic curve = 0.823). Random forest emerged as the most robust algorithm for estimating feature importance amid multicollinearity.
- We confirmed the association of well‐known chromatin state indicators—mainly DNA methylation, TEs, genes, and gene expression—in shaping recombination in *B. napus*.
- We revealed distinct recombination landscapes between the A and C subgenomes: recombination was concentrated in the subtelomeric regions of the A subgenome, whereas it was more evenly distributed along the arms of the C subgenome. These patterns aligned with differences in the distribution of chromatin state markers between the two subgenomes.

## 2 RESULTS

### 2.1 Generation of a high‐stringency recombination data set

The raw set of 171,276 COs was detected across 5132 meiotic events. Because of the very high number of individuals to be genotyped, a 15K SNP genotyping array was used that offered moderate resolution, with a median interval length of 0.87 Mbp and a mean of 2.63 Mbp (see Figure S1).

To improve data quality, we applied two stringent filters. First, plants carrying more than 100 COs—approximately 2.6 COs per chromosome per meiosis, well above the expected 1.2 CO per chromosome (Yan et al., 2023)—were removed as likely genotyping artifacts (see Figure S2). Second, COs whose intervals exceeded 2 Mbp were discarded to eliminate the long tail in the length distribution. After removing ∼13% of the original COs, these steps increased resolution (median length 0.45 Mbp, mean length 0.613 Mbp) (see Figure S3) and strengthened correlations among population‐wide recombination profiles (see Figure S4). The resulting landscapes, built with 0.3‐Mbp resolution, closely resembled those reported in earlier rapeseed maps (Yan et al., 2023), confirming the reliability of the filtered dataset.

### 2.2 Features shape the genome‐wide recombination landscape

**FIGURE 1:**
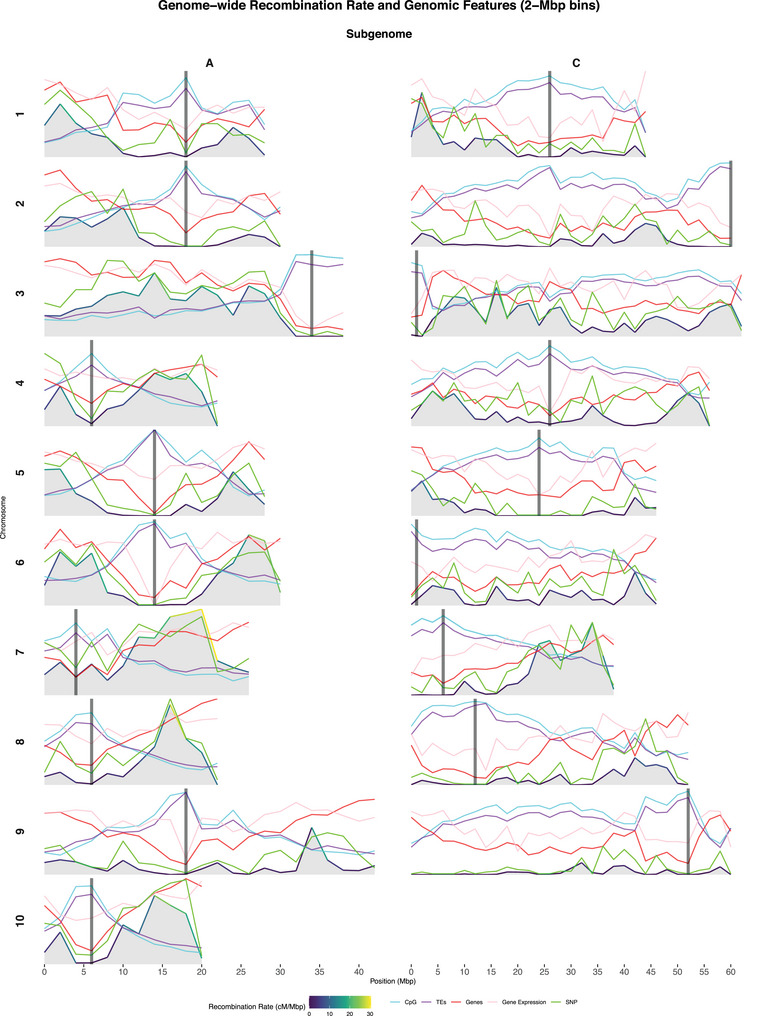
Genome‐wide recombination rate and feature values over 2‐Mbp genomic bins. The gray areas account for the recombination rate, in cM/Mbp, as well as the color of the overlying line representing its value. The features, CpG (cytosine–phosphate–guanine) methylation rate, transposable element (TE) fraction, gene fraction, gene expression level ($\mathrm{lo}g_{10}$ TPM [transcript per million] + 0.1), and single‐nucleotide polymorphism (SNP) content, are normalized by the genome‐wide maximum and represented by light blue, purple, red, pink, and green lines, respectively. Chromosomes are sorted column‐wise by subgenome and row‐wise by number. Centromere locations are indicated with thick dark gray vertical lines.

We observed strong genome‐wide associations between CO formation and epigenomic, genomic, and transcriptomic features (see Figure 1). CpG methylation levels exhibited a strong positive correlation with TEs and an inverse relationship with gene density and transcriptional activity. In rapeseed, the recombination landscape was markedly biased, with recombination rates negatively correlated with methylated, TE‐dense genomic regions, and positively correlated with gene‐rich, actively transcribed regions. The correlation study between features confirmed the trends described above (see Figure S5). COs were largely suppressed in centromeric regions, characterized by “anti‐recombinatory” feature profiles—high DNA methylation, dense TE accumulation, low gene content, and low gene expression. In contrast, recombination frequency increased progressively toward the telomeric regions, paralleling pronounced “pro‐recombinatory” feature profiles, and peaked near the chromosome ends before declining.

We observed suppression of recombination near the centromeres uniformly across chromosomes, with additional declines near telomeres. In the A subgenome, COs seem to modestly concentrate in subtelomeric euchromatin, whereas in the C subgenome, they are distributed more irregularly along the arms, yielding a mosaic landscape with no clear dominant zone. These depressions in recombination rate across the chromosome arms resemble the dips reported in the maize genome, and might harbor gene‐rich regions where low CO frequency limits selective sweeps, contributing to the retention of deleterious alleles (Rodgers‐Melnick et al., 2015).

The genome‐wide mean and median recombination rates were 5.271 and 2.32 cM/Mb, respectively. However, estimating recombination rate precisely in large populations is a complicated task, with different strategies of dealing with false positive COs (Montero‐Tena et al., 2024), which can lead to some discrepancies in estimates (M. Wang et al., 2023; Yan et al., 2023).

CO estimates are influenced by the uneven SNP density of the 15 K array. SNP‐rich windows inflate apparent recombination rates, whereas marker‐poor windows appear as recombination deserts. Normalizing CO counts by SNPs per bin preserved the genome‐wide pattern (see Figure S6), agreeing with the positive association between genetic polymorphisms and recombination (Cutter & Payseur, 2013; Hsu et al., 2022; Yang et al., 2015). We therefore excluded bins with no markers, as their zero CO rate is likely an artifact. For example, the distal end of chrC09L lacks array markers yet contains genes and shows CO activity in other studies (Yan et al., 2023). Although removing such “SNP deserts” lowers spatial resolution and may hide genuine low‐recombination domains, it yields a dataset that can be compared fairly across bins.

**FIGURE 2:**
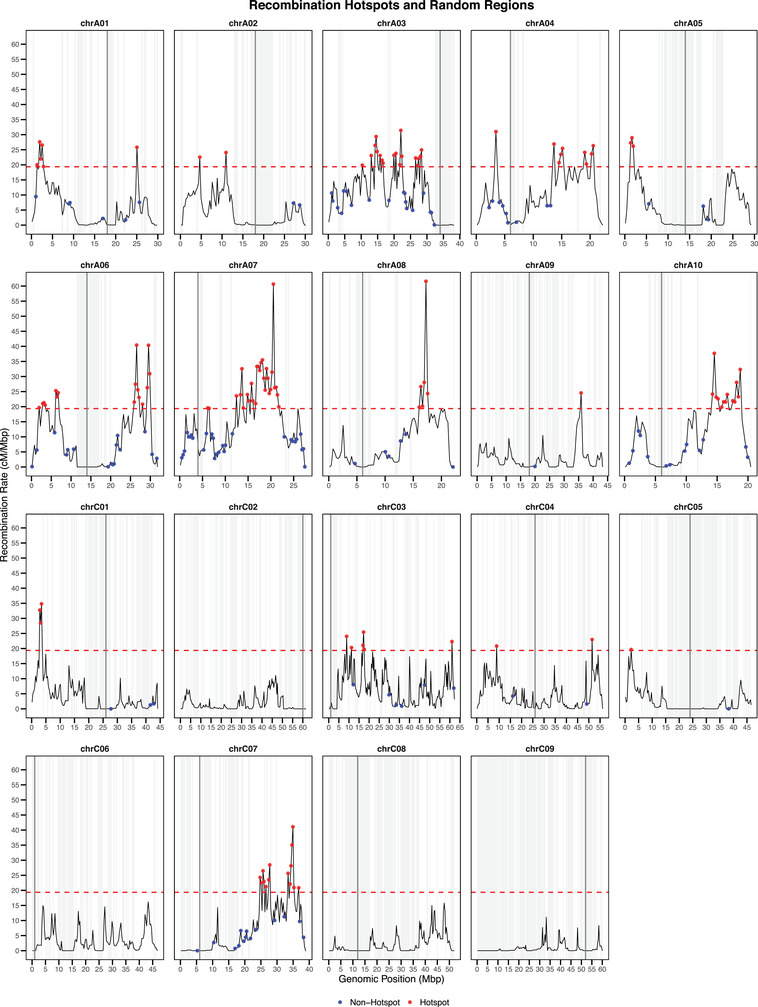
Genome‐wide distribution of hotspots and non‐hotspots regions. Hotspots (red dots) were in the upper 5% genome‐wide recombination rate, whereas non‐hotspots (blue dots) were randomly drafted from the bottom 75%, excluding single‐nucleotide polymorphism (SNP)‐empty bins. Chromosomes are labeled over the plot frames. Centromere locations are indicated with thick dark gray vertical lines. SNP‐empty bins are colored in light gray.

The highest 5% of bins displayed recombination rates at least four times greater than the genome‐wide mean, highlighting pronounced hotspots within the recombination landscape (see Figure 2). The *t*‐test confirmed the expected antagonism between pro‐ and anti‐recombinatory features in hotspots (see Figure S7). Hotspots showed significantly less DNA methylation in the CpG, CHG, and CHH contexts, and lower transposon/retrotransposon coverage than randomly sampled regions. Conversely, gene expression and gene density were higher in hotspots, and chromatin accessibility also trended upward (*P* > 0.1, not significant). Additionally, the lower values of the distance between genomic bins and the closest telomere in hotspots indicate that hotspots lie closer to telomeres, whereas non‐hotspots were drawn mainly from more centromeric regions. Together, these contrasts support a model in which COs are concentrated in hypomethylated, gene‐rich, TE‐poor euchromatic domains with accessible chromatin (Rodgers‐Melnick et al., 2015; Yelina et al., 2012, 2015).

### 2.3 Subgenome‐based differences in recombination and multi‐omic features

The A and C subgenomes exhibited distinct CO landscapes, with recombination rates that were higher in A (mean = 8.00 cM/Mbp; median = 5.31 cM/Mbp) than in C (mean = 3.54 cM/Mbp; median = 1.44 cM/Mbp), confirming earlier findings (M. Wang et al., 2023; Yan et al., 2023). Furthermore, the C subgenome contained broader nonrecombinant regions that extend beyond centromeres (see Figure 1). A similar pattern was observed for recombination hotspots, as the C subgenome only contributed with roughly a quarter of all hotspots (see Figure 2)—none on chromosomes C02, C06, C08, or C09—agreeing with the high significance level obtained by this feature in the univariate test (see Figure S7). In fact, the C subgenome yielded a similar absolute number of COs to the A subgenome despite its larger physical size (see Figure S8).

We tested if these discrepancies could be attributed to the lower SNP marker density in the C subgenome (see Figure S9)—in line with its lower genetic diversity (Qian et al., 2014). To test this hypothesis, we fitted a logistic regression model to evaluate hotspot location as a function of SNP density and subgenome identity (see Table 1). Notably, subgenome identity had a stronger effect (−1.50) on hotspot presence than SNP count (0.27), which indicates that, even after controlling for SNP density, the C subgenome is intrinsically less prone to forming hotspots.

**TABLE 1:** Logistic regression model evaluating hotspot location as a function of single‐nucleotide polymorphism (SNP) density and C subgenome identity. The table reports, for each term, the estimated coefficient, its standard error (SE), the corresponding *z*‐statistic, and the associated *p*‐value. Positive coefficients indicate an increase in the log‐odds of a bin being classified as a hotspot, whereas negative coefficients indicate a decrease. Specifically, a negative coefficient for the C subgenome reflects that, after accounting for SNP density, bins located in the C subgenome are less likely to be classified as hotspots compared with bins in the A subgenome.

Term	Estimate	SE	*z*‐score	*p*‐value
(Intercept)	−4.60708	0.24815	−18.565	<2e‐16
SNP	0.27319	0.01975	13.832	<2e‐16
C subgenome	−1.49977	0.24638	−6.087	1.15e‐09

**FIGURE 3:**
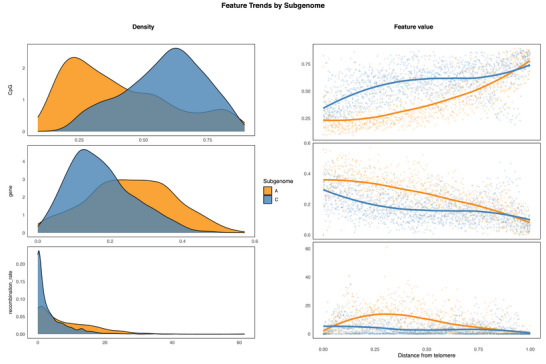
Probability density (left) and distribution across chromosome arms (right) of CpG (cytosine–phosphate–guanine) methylation rate (top), gene coverage (middle), and recombination rate (bottom) in the A (orange) and C (blue) subgenomes. Each row contains two plots corresponding to the feature indicated on the left. For each feature, both plots were derived from the same set of 0.3‐Mbp bins. In the left panels, the density curves represent each subgenome separately (A = orange, C = blue), and the *Y*‐axis shows the kernel density estimate of the feature values represented in the *X*‐axis. In the right panels, the *Y*‐axis shows the feature values, and the *X*‐axis shows the relative position along the chromosome arm (“Distance from telomere”; 0 = telomere, 1 = centromere). LOESS regression lines were fitted to the A (orange) and C (blue) subgenome values. See Figure S10 for an extended version including additional features.

Beyond differences in CO frequency, hotspot distribution, and marker density, the two subgenomes diverged sharply in the chromatin features linked to CO formation (see Figure S10). The C subgenome carried higher DNA methylation in the CpG, CHG, and CHH contexts, and greater TE coverage—features associated with recombination suppression—while the A subgenome was richer in genes and transcripts. Our observations agreed with previous findings (Z. Wang et al., 2018). These disparities generated pronounced bimodal distributions, especially for DNA methylation and gene density (see Figure 3): most C subgenome bins clustered in a high‐methylation, low‐gene mode, whereas A subgenome bins occupied a low‐methylation, high‐gene mode. These contrasts parallel the asymmetric epigenome reported by Q. Zhang et al. (2021): the A subgenome is enriched for active histone marks and shows higher gene content and transcriptional output, whereas the C subgenome is more heavily methylated, TE‐rich, and transcriptionally subdued.

Notably, features exhibit distinct patterns along the chromosome arms in the two subgenomes. In the C subgenome, variation is sharper toward the chromosome ends, whereas in the A subgenome, changes occur more gradually across the arms (see Figure S10). This pattern creates pronounced gaps, particularly in subtelomeric regions, for chromatin‐related features that diverge between subgenomes: Pro‐recombinatory features, such as genes, are enriched in the A subgenome, whereas anti‐recombinatory features, including DNA methylation and TEs, dominate in the C subgenome. These contrasting distributions likely reflect differences in recombination landscapes, with COs concentrated in subtelomeric regions of the A subgenome and more evenly distributed along the C subgenome.

### 2.4 Positive association of TE body CHH methylation rate and recombination

**FIGURE 4:**
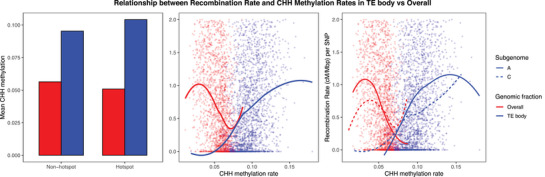
Relationship between recombination rate and CHH (where H represents any nucleotide except guanine) methylation rates in the transposable element (TE) body versus overall. Two genomic contexts are compared: All CHH's regardless of the context (overall, red) and CHH's exclusively located within TE bodies (TE body, blue). The leftmost panel shows the average CHH methylation rates, *Y*‐axis, for the bins defined as hotspots, or non‐hotspot by genomic context, *X*‐axis. The middle and rightmost panels show the relationship between normalized recombination rate per single‐nucleotide polymorphism (SNP) count (cM/Mbp), *Y*‐axis, and CHH methylation rate for each genomic context. LOESS regression lines were fit to the scatterplots. The straight line in the middle panel represents both subgenomes, whereby the straight lines correspond with the A subgenome and the dashed lines correspond with the C subgenome in the rightmost figure.

Across the genome, CHH methylation tends to occur in recombination‐poor regions, similar to CpG and CHG methylation (see Figure S5). However, when CHH sites were restricted to TE bodies, methylation was positively correlated with local CO rates, and its levels were higher (∼8%–14%) than for CHH sites across all genomic contexts (∼0%–7%), in which it was, instead, negatively correlated (see Figure 4). Despite the overall higher CHH methylation levels in the C subgenome, TE bodies exhibited slightly higher CHH methylation in the A subgenome, consistent with its enrichment in pro‐recombinatory features (see Figures S5 and S10). The results agree with findings in rice, sorghum, tomato, and maize (Peñuela et al., 2024, 2022; Rodgers‐Melnick et al., 2015), where a positive association was reported even without restricting CHH methylation to TE bodies.

Although CHH methylation represents a relatively small proportion of total methylation (Law & Jacobsen, 2010), it plays a key role in complex regulatory mechanisms that suppress TEs (Stroud et al., 2014). In maize, TEs inserted upstream of genes—often in recombination‐rich regions—are transcriptionally silenced via RNA (ribonucleic acid)‐dependent CHH methylation, forming “CHH islands” (Gent et al., 2013). In *Arabidopsis*, region‐specific CHH methylation pathways have been described: DRM2 silences short TEs at distal chromosomal ends, while CMT2 targets long, pericentromeric TEs, both acting in self‐reinforcing loops with histone modifications and small interfering RNAs (Stroud et al., 2014). Moreover, CO relocations toward distal ends have been observed in mutants lacking MOP1, a key component of the RNA‐dependent CHH methylation pathway (Zhao et al., 2021).

Taken together, these results support a model in which CHH methylation helps silence TEs located near genes in *B. napus*. Despite methylation in the CHH context, euchromatic regions remain permissive to recombination, consistent with positive associations between CHH methylation and recombination observed both in rapeseed and across other plant species.

### 2.5 Machine learning with genome‐wide data

We implemented four machine learning algorithms—regularized linear/logistic regression (LR) and three tree‐based models: decision tree (DT), random forest (RF), and gradient boosting—to identify multi‐omics predictors of recombination. Models were applied to two tasks: (1) classification, to distinguish recombination hotspots from non‐hotspot regions, and (2) regression, to predict recombination rate per genomic bin. These models were used to evaluate the predictive value of individual features and to assess consistency in feature importance across tasks and algorithms.

#### 2.5.1 Model sensitivity to multicollinearity in feature importance estimation

The recombination landscape in rapeseed is controlled by highly correlated features. As our goal was to evaluate the predictive value of recombination rate and hotspot distribution among these features, presumed to contribute similarly to the model's predictions, we inspected the robustness of different machine learning models under clusters of colinear features (see Section 5). Among all models tested, RF consistently produced the most stable rankings of feature importance, maintaining over 97% Spearman correlation between rankings obtained with and without highly correlated features (see Table 2). RF exhibited the lowest intra‐cluster variability in importance scores among correlated features, indicating a more balanced attribution of predictive power across colinear variables. Additionally, the correlation of RF in feature rankings between both tasks, classification and regression, was very high compared to the other models, with around 55% for all features and 80% for the best representative per cluster (see Figure S11). Similarly, RF feature rankings were highly consistent within the same task when compared to other tree‐based models (see Figure S12). Owing to its capacity to model nonlinear relationships, straightforward interpretability, and strong performance in our analyses, we selected RF as the reference model for assessing feature importance in both classification and regression tasks.

**TABLE 2:** Consistency of feature importance rankings under multicollinearity across four algorithms. Spearman correlation coefficients were calculated using the best representative per cluster between rankings of models developed with all features or with only the best representative per cluster of colinear features. The table also reports the average standard deviation (SD) of feature importance within the same clusters, the average SD across all features, and the ratio between these two values.

Task	Model	Spearman rank correlation	Average within‐cluster SD	Overall feature SD	SD ratio (within/overall)
Classification	Random forest	1.00	2.21	4.86	0.455
Classification	Boosted trees	0.810	0.0645	0.0827	0.780
Classification	L reg	0.548	0.310	0.413	0.751
Classification	Decision tree	0.548	3.71	14.8	0.250
Regression	Boosted trees	0.976	0.0206	0.0544	0.378
Regression	Rand forest	0.976	392	1140	0.343
Regression	Decision tree	0.810	1180	12000	0.0975
Regression	L reg	0.667	0.450	1.16	0.387

#### 2.5.2 Model performance

Hotspots were predicted with great performance. The RF achieved a mean fold‐wise area under the receiver operating characteristic curve (AUROC) value of 0.858 (see https://jamonterotena.github.io/bnapus.reco.ml/)—the best among classifiers. The overall AUROC was 0.823 (see Figure S13), obtained with the probabilities for all original values and their corresponding predictions obtained during cross‐validation.

**FIGURE 5:**
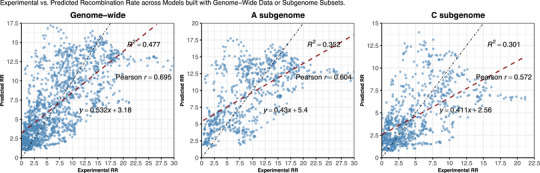
Scatterplot with regression line of experimental and predicted recombination rate (RR) values, in cM/Mbp, based on the random forest models built with genome‐wide (left), A subgenome (middle), or C subgenome (right) data. *R* ^2^, Pearson *r*, and slope‐intercept equations are shown.

**FIGURE 6:**
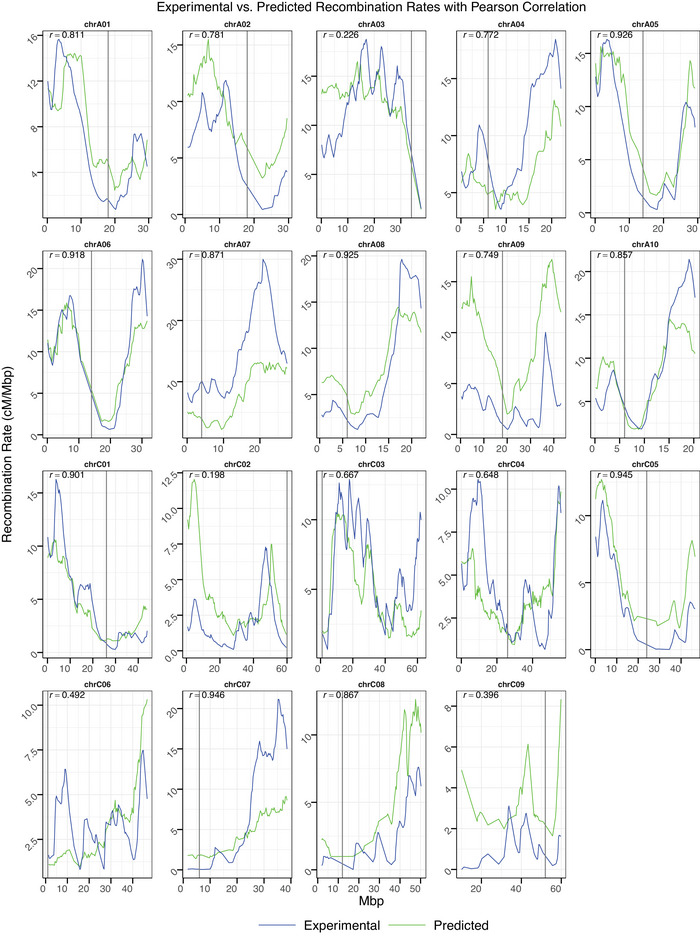
Genome‐wide distribution of the actual and predicted values of recombination rate, in cM/Mbp, over 0.3‐Mbp genomic bins. Actual and predicted values are indicated by blue and green lines, respectively. Plots are titled with chromosome names. Straight vertical black lines represent centromere locations.

Recombination rate was predicted via regression with moderate performance. RF yielded a mean fold‐wise $R^{2}$ of 0.662 (see https://jamonterotena.github.io/bnapus.reco.ml/) and an overall $R^{2}$ of 0.477, obtained by comparing all the original values with their corresponding predictions (see Figure 5). Nevertheless, predicted versus observed rates were strongly correlated within chromosomes (mean Pearson ρ = 0.731), reaching ∼0.90 on C07, C05, A05, and A08 but dropping below 0.50 on C02, A03, C09, and C06 (see Figure 6; see https://jamonterotena.github.io/bnapus.reco.ml/). Thus, although absolute rate estimates were imperfect, the model captured the broad recombination trends along most chromosomes.

#### 2.5.3 Feature importance

**FIGURE 7:**
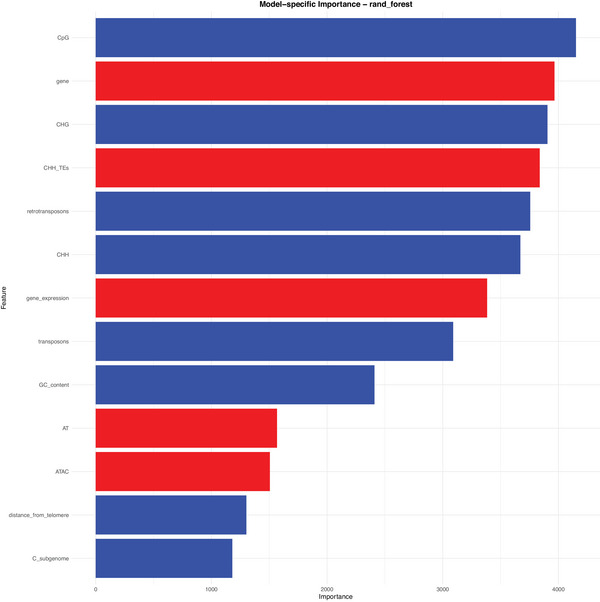
Feature ranking by decreasing Gini impurity‐based importance scores obtained by the random forest regression models predicting recombination rates with genome‐wide feature data. Features positively correlated with recombination are in red, whereas the negatively correlated ones are in blue. ATAC, assay for transposase‐accessible chromatin; CpG, cytosine–phosphate–guanine; G (in CpG and CHG), guanine; GC content, guanine–cytosine content; H (in CHH and CHG), any nucleotide except guanine; TE, transposable element.

In the RF regression, CpG methylation was the strongest predictor of CO rate (see Figure 7; see Figure S14). It was followed closely by highly correlated features—gene fraction; CHG, CHH, and TE‐body CHH methylation; and retrotransposon coverage, gene expression, and transposon coverage. Heterochromatin is enriched for TEs that are transcriptionally silenced by dense CpG/CHG methylation, which is maintained with MET1 or CMT3, respectively. These methyltransferases reinforce marks such as H3K9me2 via RNA‐dependent DNA methylation, which compact chromatin and suppress COs (Stroud et al., 2012; Yelina et al., 2015). By contrast, recombination‐prone euchromatic arms are TE‐poor, transcriptionally active, and hypomethylated, except for local CHH peaks that silence individual TE bodies (Rodgers‐Melnick et al., 2015). Because these features colocalize, their pairwise correlations are intrinsically high, and the RF model distributes importance almost interchangeably across them. Nonetheless, CpG methylation claims the top rank—probably reflecting its dual role as the most direct indicator of TE silencing and the methylation context most tightly linked to CO suppression. Collectively, these variables provide integrated proxies for local chromatin state, the principal determinant of the recombination landscape (Hsu et al., 2022).

In classification, feature importance rankings obtained closely resembled those obtained in the regression analysis (see Figures S15 and S16). Although colinear features ranked on top, the gap between the leaders and the rest widened. CHG methylation received at least twice the importance score of any feature other than CpG methylation. This suggests that hotspots can be distinguished almost entirely by a narrow set of signals, so adding other, closely related variables yields little additional predictive power.

Telomeric proximity climbed up to the second position in classification, even though it ranks among the weakest predictors of absolute recombination rate. Its prominence reflects how the data were sampled: hotspots fall mainly in subtelomeric euchromatin, whereas non‐hotspots were drawn largely from pericentromeric heterochromatin (see Figure 2). As a continuous predictor of CO rate, however, telomeric distance loses strength because centromere coordinates are approximate, SNP density is uneven, bins lacking markers were excluded, and the two subgenomes are equally represented, as the C subgenome could have a different recombination landscape, diluting any genome‐wide correlation with recombination.

Retrotransposons obtained notably higher importance scores in predicting recombination rate than DNA transposons, even when the two categories are present in comparable numbers. One plausible reason is structural: LTR (long terminal repeat)‐Gypsy and LTR‐Copia elements—which constitute the bulk of the *B. napus* repeatome (Lee et al., 2020)—cluster in a high number of copies, forming large, dense heterochromatin regions (Wei et al., 2013). At the same time, low copy number Copia/Gypsy families are the main CHH‐hypermethylated TEs within 1 kb upstream of genes in maize that coincide with CO hotspots (Gent et al., 2013). Because retrotransposons can mark both large heterochromatic blocks and gene‐proximal CHH islands, their genomic coverage may capture recombination‐relevant chromatin states more faithfully than the shorter, more scattered DNA‐TEs.

#### 2.5.4 Interaction analysis

After assessing the predictive power of features, we next asked whether their effects are simply additive or if they interact. Using the RF regression predictions, we assessed how much the interactions between predictors contribute beyond their individual main effects with Friedman's *H*‐statistic (Friedman & Popescu, 2008). An *H* value of 0 signifies that interactions between features add no extra predictive information beyond their individual effects, whereas an *H* of 1 means the model's signal for those variables is explained entirely by their joint effect, leaving no independent main effects.

Most features showed moderate interaction strengths: on average, the overall interaction of features with every other feature combined explained around 9% of the model's variance (see Figure S17). One pair, however, stood out. Subgenome identity interacted strongly with telomeric distance, accounting for ∼50% of their combined contribution to predicted CO rate, suggesting that contrasting feature trends observed between the A and C subgenomes (see Figure 3; see Figure S10) significantly shape distinct recombination landscapes between the rapeseed subgenomes.

### 2.6 Machine learning with single‐subgenome input

Given the distinct recombination landscapes of the A and C subgenomes, we asked whether models built and validated solely on one subgenome would outperform those derived from the opposite subgenome or from the genome‐wide dataset. Subgenome‐specific models may also uncover feature effects that are unique to each subgenomic context.

The RF regression model developed with A subgenome data performed better (mean fold‐wise $R^{2}$ = 0.597, overall $R^{2}$ = 0.352, mean chromosome‐wise Pearson ρ = 0.780) than its counterpart derived from the C subgenome (mean fold‐wise $R^{2}$ = 0.515, overall $R^{2}$ = 0.301, mean chromosome‐wise Pearson ρ = 0.678) (see https://jamonterotena.github.io/bnapus.reco.ml/; see Figure 5). Although the A‐specific $R^{2}$ is lower than that of the genome‐wide model (see Figure 5), its higher chromosome‐level correlation shows that it captures spatial trends more faithfully (see https://jamonterotena.github.io/bnapus.reco.ml/). Nearly identical feature‐importance profiles in the two subgenomic models indicate that homoeologous chromosomes encode similar recombination patterns, differing mainly in regional signal strength and noise (see Figures S18 and S19).

Accumulated local effect (ALE) curves measure how a single predictor alters the model's prediction across local intervals while averaging the local values of all other variables, therefore constituting a multicollinearity‐robust approach to estimate main effects. Positive ALEs on an interval indicate that the feature raises the predicted recombination rate when moving across the interval, while negative main ALEs represent lowering effects. Overall, ALE profiles correlated well across subgenomes and matched the expected relationship with recombination (see Figures S20 and S21).

**FIGURE 8:**
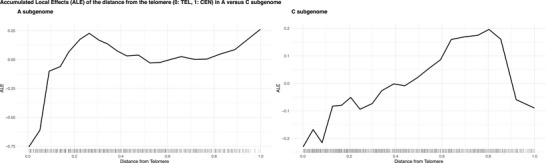
Accumulated local effect (ALE) curves of distance from the telomere to the 0.3‐Mbp bins in the models built on A subgenome data (left) and C subgenome data (right). The *X*‐axis represents the distance from the telomere, ranging from 0 (telomere) to 1 (centromere), whereby the *Y*‐axis indicates the ALE, centered around 0. The distributions of bins across the feature values are shown with rugs alongside the *X*‐axis.

However, ALE profiles for telomeric proximity differed sharply between subgenomes (see Figure 8). For the A subgenome, the most positive local effects appeared in the subtelomeric regions (∼0.25 relative distance from the telomere), where the telomeric proximity increased the predicted recombination rate by up to +0.25. In the C subgenome, the peak shifted inward: pericentromeric segments (0.70–0.80) raised the rate by up to +0.20. Telomeric ends produced the most negative effects in both subgenomes (−0.75 in A, −0.20 in C). Centromeres were strongly suppressive in C but showed a slight positive artifact in A, likely caused by sparse data after excluding SNP‐empty bins.

Taken together, our results show that the two parental subgenomes of rapeseed carry distinct recombination landscapes. In the A subgenome (*B. rapa*), COs cluster toward subtelomeric regions; in the C subgenome (*B. oleracea*), they concentrate in pericentromeres. This pattern mirrors earlier genetic maps (Yan et al., 2023), where chromosomes C06, C08, and C09 exhibit elevated pericentromeric recombination, whereas their A‐homoeologs peak near the telomeres despite being physically shorter. Telomeric distance, however, explains only a small fraction of the variance in CO rate; the dominant predictors in both subgenomes are the same chromatin‐state markers that govern recombination genome‐wide.

## 3 DISCUSSION

We built machine learning models that accurately predicted recombination rate and hotspot location and classified multi‐omic features according to their predictive power. We conducted unconventional machine learning techniques, such as the application of ALE curves, to understand the effect that local values had on the target both for single features and combination of feature values. This complements the general effects represented by importance scores, representing a model‐agnostic approach to understand model predictions that is strongly robust to multicollinearity. Because colinear variables are commonplace in biological studies and complicate the estimation of their actual contribution to explaining biological phenomena, we implemented a statistical comparison of results in the presence or absence of multicollinear features across the four algorithms to select the model most consistent in the face of highly correlated features. The RF was the most consistent model under multicollinearity, reflecting the enhanced robustness of tree‐based models compared to LR models (see Figure S12). We encourage researchers to incorporate this test when selecting algorithms to ensure fair comparisons among predictors.

The main limitation of this study was the coarse resolution of the inferred CO intervals—median length of 0.45 Mbp (see Figure S1)—mainly due to the low diversity among the founders selected for developing the two large multiparental populations (Krenzer et al., 2024) and the application of the 15K SNP chip. Clustering CO events into contiguous 0.3‐Mbp windows reduced the resolution for pinpointing recombination breakpoints, hindering efforts to investigate local factors that shape recombination at a fine scale or to identify novel features associated with recombination patterns, such as DNA motifs (Wijnker et al., 2013). Additionally, the sparse polymorphism characterizing genotyping array can limit both the power and precision of recombination estimates (Scott et al., 2020) due to the inability to detect double COs or structural variants located within longer intervals between informative markers. Furthermore, the selection of markers in predefined loci can cause the underestimation of recombination rates in SNP‐poor regions and the overrepresentation of highly polymorphic regions, leading to ascertainment bias (Albrechtsen et al., 2010). Nevertheless, whereas most studies use whole genome sequencing genotyping (Demirci et al., 2017; Peñuela et al., 2022; Si et al., 2015) or 60K SNP arrays (Boideau et al., 2022; M. Wang et al., 2023; Yan et al., 2023) in small sets of recombinant inbred lines, we analyzed two outbred populations of ∼1000 plants, detecting far more COs, and thus bringing recombination‐rate estimates closer to reality—after stringent haploMAGIC filtering to remove false positives. Because CO calls scale with marker density, recombination rates can be confounded by local SNP content; however, after normalizing for SNP density, rates remained highly correlated with the raw values, reflecting evidence that hotspots coincide with regions of elevated polymorphism. To avoid such confounding, we excluded SNP density from the predictor set.

The features that indicate chromatin state, well‐known to shape recombination globally, are also the major players in *B. napus*, suggesting conservation of the main recombination mechanisms. These factors are highly correlated among each other, and their tight link with recombination was clear: Proxies for chromatin state modulated CO formation throughout recombination maps (see Figure 1) and consistently obtained highly significant *p*‐values in the *t*‐test (see Figure S7) and the highest importance scores by the RF in predicting recombination rates (see Figure 7) and hotspot locations (see Figure S15).

Our results indicate that subgenomes have distinct recombination landscapes in *B. napus* (see Figure 8). The A subgenome is subtelomeric‐dominant, whereas the C subgenome is pericentromeric‐dominant. Besides, the C subgenome was importantly less recombination‐prone. These different recombination patterns concord with contrasting feature patterns: The feature profile of the C subgenome—higher for anti‐recombinatory factors and lower for the pro‐recombinatory ones—evolves differently along chromosome arms, which reflects their distinct recombination landscape (see Figure 3; see Figure S10). Post‐hybridization epigenomic remodeling could have contributed to reshaping the recombination landscape across subgenomes. Some studies reported changes in epigenomic features affecting newly resynthesized *B. napus*, for example, loci frequently displaying different DNA methylation patterns to the progenitor genomes (Lukens et al., 2006). In polyploids, these epigenomic modifications have implications in gene expression (Song & Chen, 2015) and genomic stability. Lower recombination in the C subgenome, combined with breeding selection and asymmetric introgression favoring *B. rapa* over *B. oleracea*, could have contributed to reducing its genetic diversity, evident in stronger linkage disequilibrium (Qian et al., 2014) and lower SNP density (see Figure S9).

Our data also show that telomeres display lower recombination, while centromeres lack it altogether (see Figures 1 and 8). Although recombination is generally thought to be high in telomeres, this pattern matches observations in maize, where COs peak in subtelomeric regions and decay before the chromosome ends (Rodgers‐Melnick et al., 2015). Because reduced DNA exchange makes these regions less prone to selective sweeps, they may carry a higher genetic load. We therefore recommend breeding strategies aimed at unlocking the latent recombinogenic potential of telomeres in *B. napus*.

Our results confirm the positive association of recombination and CHH methylation within TE bodies in rapeseed, which also display pronounced hypermethylation compared to other genome fractions. Additionally, positive correlations are observed for TE‐body CHH methylation with genes and gene expression (see Figure S5). These observations support the idea that, inside recombination hotspots, methylation of CHH islands marks gene‐proximal TE bodies strongly, preventing their activation via RNA polymerase‐mediated transcription (Gent et al., 2013). This colocalization of TE‐body CHH methylation with CO sites does not necessarily indicate that CHH methylation promotes recombination, as contrasting patterns have been reported in *Arabidopsis* and maize CHH methylation mutants (Christophorou et al., 2020; Zhao et al., 2021). CHH methylation within TE bodies negatively correlates with TE coverage per bin, especially in retrotransposons (see Figure S5), which is consistent with the finding that most gene‐proximal LTR retrotransposons are low copy (Grandbastien, 2015). Previous studies reported positive relationships between CHH methylation and recombination without restricting to TE bodies (Peñuela et al., 2024, 2022; Rodgers‐Melnick et al., 2015). In *B. napus*, the positive correlation observed specifically in TE bodies could be explained by the pronounced separation between genes and TEs, evidenced by the high negative correlation (−0.76 with retrotransposons, −0.49 with transposons; see Figure S5), which could affect the CHH methylation signal at the resolution applied in this study. In *Arabidopsis*, a similarly contrasting distribution between TEs and genes (X. Zhang, 2008) could explain the weak overall positive association between CHH methylation and CO frequency (Peñuela et al., 2024). Overall, TE‐body methylation of CHH islands and its association with recombination are complex biological processes, which could follow species‐dependent trends (Martin et al., 2021).

We observed that the coverage with ATAC‐accessible regions was positively related to CO formation (see Figure 7). However, we expected this feature to achieve better predictive scores since other features that are universally clearly linked with chromatin accessibility were consistently among the best predictors. This could be due to the dilution of the ATAC‐seq (assay for transposase‐accessible chromatin using sequencing) signal. We could not observe clear relationships with nucleotide‐based features at this resolution of the recombination data. Therefore, a couple of questions remain unresolved: Do hotspots in the rapeseed genome exhibit higher GC content as a result of GC‐biased gene conversion (Rodgers‐Melnick et al., 2015), or are they instead enriched for A/T‐rich sequences typical of gene promoters (Wijnker et al., 2013)?

## 4 CONCLUSIONS

Taken together, our findings show that the principal determinants of COs are conserved in *B. napus*, explore patterns of multi‐omics features previously associated with recombination, and propose a model in which the recombination landscape differs in the two sub‐genomes. The subgenome‐specific patterns could have an impact on allele recombination during breeding and future efforts to engineer increased recombination rates or altered recombination patterns.

Beyond meiotic recombination, the rapeseed genome is characterized by homoeologous exchanges (HEs), that is, structural rearrangements between the two subgenomes. Together with meiotic COs, HEs have the potential to unlock the breeding potential of the C subgenome. Consequently, investigating the landscape of HEs and how epigenomic remodeling within these regions—due to asymmetric epigenomes (Zanini et al., 2025; Q. Zhang et al., 2021)—influences phenotypic variation represents a promising direction for future research.

## 5 MATERIALS AND METHODS

### 5.1 Sequencing data

*B. napus* plants belonging to two large multiparental populations were genotyped using a 15K SNP array (Clarke et al., 2016). These populations, consisting of 1573 and 1550 individuals for Populations 1 and 2, respectively, originated from two panels of 50 elite German varieties and were developed through four generations of outcrossing following a simulation‐based crossing program aimed toward maximizing general combining ability while preserving genetic diversity as described by Krenzer et al. (2024). After processing raw SNP data outlined in “Section 5” of Abdollahi et al. (2024), a final dataset of 11,443 SNP markers was retained across all 19 chromosomes of *B. napus* (AACC, 2*n* = 4*x* = 38). Note that 2566 individuals were retained—1243 and 1323 in Populations 1 and 2—after discarding plants with missing pedigree information about the founder lines.

### 5.2 Generation of recombination data

We implemented haploMAGIC (Montero‐Tena et al., 2024) for detecting recombination events across the two parental haplotypes of the individuals spanning generations G2 to G4. In these generations, the offspring inherited their haplotypes from recombination‐informative meioses occurring in heterozygous parents. haploMAGIC reconstructs parental haplotype sequences using pedigree information by resolving diploid SNP genotypes into phased grandparental haplotype blocks. These blocks become fixed along chromosomes after meiotic recombination, enabling the identification of COs within intervals where transitions between parental haplotypes occur. To address generation‐specific genotyping error patterns, we configured haploMAGIC with the options min = 2/5/3 for generations G2, G3, and G4, respectively, along with imp = imputeTHonly and cor = correctFalseHom. These settings ensured consistent genome‐wide recombination rate trends across generations (see Figure S22). A total of 171,276 CO intervals were identified across all informative meioses (median length = 874,808 bp, mean length = 2,626,983 bp) (see Figure S1). The link to the raw CO set can be found in the “Data Availability Statement” section.

We performed two quality filters on the CO set by (1) removing COs from outlier individuals having more than 100 COs over all meiotic events (see Figure S2), and (2) discarding CO intervals longer than 2 Mbp. The filtered CO set consisted of 148,600 COs (median length = 444,895 bp, mean length = 612,933 bp) (see Figure S3).

### 5.3 Calculation of recombination rate

Recombination rates were first estimated by counting the number of CO events occurring within nonoverlapping 0.3‐Mbp bins across the genome. When a CO interval spanned multiple bins, its contribution was proportionally distributed based on the fraction of the interval overlapping each bin. Recombination rate in cM/Mbp was then calculated for each bin using the following formula:

$$\;\mathrm{Recombination}\;\mathrm{rat}e_{i}=\frac{100\times \left(\frac{\mathrm{Crossovers}\;\mathrm{in}\;\mathrm{bin}\;i}{\mathrm{Informative}\;\mathrm{meioses}}\right)}{\mathrm{Bin}\;\mathrm{length}\;\mathrm{Mbp}}$$

The number of informative meioses varied by population and was equal to twice the number of informative individuals retained after filtering individuals: 1157 for Population 1 and 1265 for Population 2.

### 5.4 Sampling of hotspot and non‐hotspot regions

Hotspot bins were defined as those falling within the top 5% of recombination rates across the genome. For each chromosome, we randomly selected the same number of non‐hotspot bins (with recombination rate below the 75th percentile, excluding SNP‐empty bins) as hotspot bins (above the 95th percentile), thereby ensuring balanced class sizes per chromosome while maximizing the contrast between the two groups (see Figure 2).

### 5.5 ATAC‐seq, RNA sequencing, and whole‐genome bisulfite sequencing data analysis

Data were obtained from Zanini et al. (2025) and included the following: ATAC‐seq and RNA sequencing data for 3.5‐week‐old leaves, 9‐day‐old roots, immature siliques, 3‐day‐old seedlings, immature flower buds of rapeseed, and whole‐genome bisulfite sequencing (WGBS) data for the first three tissues.

Data processing and analysis was carried out as described by Zanini et al. (2025), with the following deviations:

- All reads from the three datasets were aligned to the original *Express 617* v1 reference genome (Lee et al., 2020)
- Transcript abundance quantification was done with kallisto v0.50.0 (Bray et al., 2016).
- All cytosines were included, regardless of their coverage with WGBS reads.

### 5.6 Extraction and calculation of features

#### 5.6.1 Annotation‐based features

Genetic features were incorporated from the *Express 617* genome annotation (Lee et al., 2020). TEs were annotated using the EDTA pipeline (Su et al., 2021), which was provided with a custom TE library. Repeats were subsequently masked using RepeatMasker (Smit et al., 2013–2023). Where genomic intervals overlapped between feature types, overlapping regions were trimmed to avoid double‐counting.

Transposons were defined as the aggregated fractions of the DNA transposon families DNA/DTA (hAT transposons), DNA/DTC (CACTA transposons), DNA/DTH (Helitron transposons), DNA/DTM (Mutator transposons), DNA/DTT (Mariner/Tc1 transposons), and DNA/Helitrons. Retrotransposons included LTR/Copia, LTR/Gypsy, and LTR/Unknown elements. The overall TE category comprised all annotated TE families.

For each genomic bin, we calculated the proportion (range 0–1) of the genomic bin's length that was covered by each annotation feature.

#### 5.6.2 DNA methylation

WGBS data were processed beginning with adapter and quality trimming using TrimGalore 0.6.7 (https://github.com/FelixKrueger/TrimGalore) with the parameters –paired –clip_R1 8 –clip_R2 8 –three_prime_clip_R1 8 –three_prime_clip_R2 8. Trimmed reads were aligned to the *Express 617* v1 reference genome using Bismark 0.24.0 (Krueger & Andrews, 2011). Duplicate reads were removed using the deduplicate_bismark script, and methylation calls were extracted for CG, CHG, and CHH contexts using bismark_methylation_extractor.

Methylation reports were converted into bedGraph format using bismark2bedGraph for downstream processing. Genomic interval coordinates were adjusted to 1‐based indexing, and calls from top and bottom strands were merged. For each cytosine position, the single‐base methylation rate was calculated as the ratio of methylated calls to total calls. Cytosines not called in all replicates were excluded. For each tissue, methylation rates were averaged across replicates, and final values were obtained by averaging across all tissues. The final feature values represent average single‐base methylation rates per bin for each context: CpG, CHH, and CHG.

TE body CHH methylation (CHH_TEs) represents the average single‐base methylation rate of cytosines in the CHH context that are located within any annotated TE of each bin. To obtain the overlap of cytosines and TEs, we applied the intersect function of BEDTOOLS 2.27.1 (Quinlan & Hall, 2010).

We compared the single‐base methylation rates with weighted methylation rates calculated as the total number of methylated reads divided by the total number of reads per bin. Weighted values were calculated by summing calls from all tissues and replicates and applying the formula reported in Schultz et al. (2012). The two methods produced nearly identical results, indicating that coverage biases had minimal impact on methylation estimates (see Figure S23).

#### 5.6.3 Dinucleotides

We applied the scripts used in Demirci et al. (2018) to calculate the proportion of the bins covered by each dinucleotide.

#### 5.6.4 GC content

We used the function nuc of BEDTOOLS 2.27.1 (Quinlan & Hall, 2010) to estimate the proportion of guanine and cytosine bases per bin.

#### 5.6.5 Distance from the telomere

We inferred the locations of centromeric regions in the *Express 617* genome (Lee et al., 2020) using the centromere interval coordinates reported in Mason et al. (2016) for the *Darmor* reference genome (Rousseau‐Gueutin et al., 2020). Centromeric sequences were extracted from the *Darmor* v10 assembly and aligned to the *Express 617* v1 genome using NUCMER 4.0 (Marçais et al., 2018) with the options –maxmatch –nosimplify to retain repetitive regions. The resulting alignments were filtered using the delta‐filter with the options ‐r ‐q to retain only the best global hits. Aligned regions consistently clustered in specific genomic areas, allowing us to define a single representative centromeric position per chromosome. In cases where multiple distinct alignment blocks were observed, we prioritized those closest to the centromeric intervals defined by Mason et al. (2016).

For each bin, we calculated its relative position along the chromosome arm as the distance from the centromere to the bin midpoint divided by the distance from the centromere to the closest telomere. This ratio ranges from 0 to 1 and reflects how close a bin is to the centromere versus the telomere.

#### 5.6.6 Chromatin accessibility

Raw reads were trimmed using cutadapt 4.0 and aligned to the *Express 617* v1 reference genome with Bowtie2 2.4.5 using the parameters ‐k 10 ‐X 1000 –very‐sensitive. Open chromatin regions (OCRs) were identified using Genrich in ATAC‐seq mode with the options ‐j ‐d 150 ‐y ‐a 200 ‐r.

For each bin, we calculated the overlap with OCR in each tissue individually. Given the strong tissue specificity of chromatin accessibility, the tissue‐level coverage values were first transformed before being averaged across tissues using the following formula:

$$\frac{\mathrm{lo}g_{10}\left(\mathrm{accessible}\;\mathrm{length}+1\right)}{\mathrm{lo}g_{10}\left(\mathrm{total}\;\mathrm{bin}\;\mathrm{length}\right)}$$

This transformation constrained the values between 0 (completely inaccessible) and 1 (fully accessible) and reduced the skew from large tissue‐specific peaks while preserving relative accessibility information across bins.

#### 5.6.7 Gene expression

After quantifying transcript abundance with kallisto v0.50.0 (Bray et al., 2016) using the *Express617* v1 reference, we averaged the transcript per million (TPM) of genes across replicates of each tissue. To account for tissue‐specific differences in gene expression, we applied logarithmic transformation to TPM values prior to averaging across tissues using the following formula:

$$\mathrm{lo}g_{10}\left(\mathrm{TPM}+0.1\right)$$

After intersecting these values with the feature table, the gene expression level was calculated as the average transformed TPM among the genes within each bin.

#### 5.6.8 Subgenome

A binary feature, *C_subgenome*, was created with the values TRUE/1 for bins annotated in the subgenome C (chrC01‐chrC099) or FALSE/0 for bins in the subgenome A (chrA01‐chrA10).

#### 5.6.9 Single‐nucleotide polymorphisms

The genomic positions of the SNPs used for CO detection were used to count the number of SNPs within each bin.

### 5.7 Construction of feature tables

A feature table was constructed for each machine learning task, with bins represented as rows and the following columns: bin coordinates (chromosome, start, and end base pairs), target value, and feature values. The target variable depended on the task: for classification, the target was a binary variable indicating hotspot presence (TRUE/1 for hotspot bins, FALSE/0 for non‐hotspots); for regression, the target was the normalized recombination rate (a continuous numeric value). Feature values were computed per bin using the intersect function of BEDTOOLS 2.27.1 (Quinlan & Hall, 2010), implemented via a custom script (https://github.com/jamonterotena/Custom‐BEDTools‐intersect), which extracted all feature elements overlapping with the bin intervals. The link to the feature table can be found in the “Data Availability Statement” section. We checked that all genomic intervals were in 1‐based indexing before processing.

### 5.8 Two‐sample statistical tests

We applied a two‐sample Student's *t*‐test with equal variance to compare the means of each feature between hotspot and non‐hotspot regions. The two‐sample chi‐square test for categorical values was applied to test differences between the number of hotspots between the A and C subgenomes. The null hypothesis (*H*_0_) assumed no significant difference in the mean values of a given feature between the two groups, while the alternative hypothesis (*H*_1_) proposed that a significant difference existed. In order to meet the assumptions of the *t*‐test, we normalized them by subtracting the mean and dividing by the standard deviation (SD). To account for the increased probability of false positives due to multiple comparisons, we applied the Bonferroni–Hochberg correction to adjust the *p*‐values and control the false discovery rate. Features with adjusted *p*‐values below 0.1 were considered significant.

### 5.9 Logistic regression analysis of hotspot location

We fitted a generalized linear model with a binomial error distribution using the glm function from the R package stats (R Core Team, 2024). Statistical significance was assessed using Wald *z*‐statistics and associated *p*‐values. The model was specified as follows:

hotspot∼SNP+C_subgenome

where the response variable indicates whether a genomic bin qualifies as a hotspot. Positive coefficients correspond to an increased probability of hotspot occurrence, whereas negative coefficients indicate a decreased probability.

### 5.10 Simple exponential smoothing

Before inputting the data for training predictive models, exponential smoothing with alpha = 0.1 was applied using the function ses of the R package forecast 8.24.1 (Hyndman & Khandakar, 2008) to the recombination rate values and the feature values to remove noise associated with the abrupt change in adjacent windows. We did not smooth features in which genome splitting could not cause noise, that is, the distance from the telomere and C subgenome.

### 5.11 Machine learning modeling

Before modeling, we grouped homoeologous chromosomes together during cross‐validation in order to prevent data leakage from shared evolutionary history. Chromosome A10, which lacks a homoeologous counterpart in the *B. napus* genome, was grouped with chromosomes A09 and C09 due to their reported chromosomal synteny (Higgins et al., 2018). This resulted in a ninefold cross‐validation strategy, where each chromosome group served as the validation set in onefold, while the remaining groups were used for training. Homoeologs were replaced by chromosomes when models were developed using data from a single subgenome. Bins were shuffled within chromosomes to remove positional dependencies and ensure that models relied solely on feature values. During each fold of cross‐validation, data preprocessing was applied to the training set, including conversion of categorical variables into dummy variables, removal of predictors with zero variance, and normalization of all numeric predictors. Model parameters were then tuned using Bayesian optimization to improve predictive performance, and models were evaluated on the validation fold using metrics appropriate to the task.

Machine learning models were developed to classify recombination hotspots and to predict normalized recombination rates. Modeling was performed in R using the tidymodels framework (Kuhn & Wickham, 2020). For each task, we trained four algorithms: DTs, regularized logistic (or linear) regression (LR), RF, and boosted trees (BT). Hyperparameters were optimized for each model type. DT models included tuning of cost complexity, tree depth, and minimum node size. LR models were tuned for penalty and mixture parameters. RF models were optimized for the number of predictors sampled at each split (mtry), number of trees, and minimum node size. BT models were tuned for mtry, number of trees, learning rate, tree depth, loss reduction, and minimum node size. For classification, the model achieving the highest average receiver operating characteristic area under the curve across cross‐validation folds was selected. For regression, the best model was selected based on the highest average $R^{2}$. Additionally, Pearson's correlation coefficient was calculated by chromosomes between predicted and actual values.

### 5.12 Feature importance estimation

To assess the contribution of individual features toward predicting hotspots or recombination rate, we evaluated model‐specific importance metrics. In DT and RF models, importance was quantified as the total reduction in Gini impurity (for classification) or in residual sum of squares (for regression) associated with splits on each feature. In BT models, importance was measured as the total gain, representing the cumulative improvement in the loss function attributed to each feature across all trees. For LR models, feature importance was derived from the absolute value of standardized coefficients, reflecting the magnitude and direction of each predictor's contribution after penalization.

Feature direction was derived differently for linear and tree‐based models. For LR models, direction corresponded to the sign of the standardized coefficient, indicating whether the predictor increased (positive sign) or decreased (negative sign) the likelihood of hotspot occurrence or recombination rate. For tree‐based models, direction was inferred post hoc: in classification tasks, it was based on whether the mean feature value was greater in predicted hotspot bins than in predicted non‐hotspot bins, while in regression tasks, it was determined by the sign of the Spearman correlation between feature values and the model‐predicted recombination rate.

### 5.13 Model selection

Among all the algorithms tested, we selected a model for further analyses based on two criteria: (1) its predictive performance, determined by the best results obtained during Bayesian hyperparameter optimization, and (2) the robustness of its feature importance rankings under multicollinearity.

We assessed this robustness by comparing models trained on two feature sets: one containing all features and another containing a single representative per predefined feature cluster. Clusters grouped highly correlated features (Pearson correlation coefficient > 0.80) (see Figure S5), including one cluster with general methylation rate features (CpG, CHG, CHH) and annotation‐based features (gene and retrotransposon coverage), and another cluster comprising nucleotide‐based features such as GC content and AT dinucleotide (adenine–thymine dinucleotide) frequency. In the reduced feature set, we retained only the representative of each group—namely, CpG for methylation/annotation‐based features and AT for nucleotide composition—instead of all correlated features.

For each combination of model, feature set, and machine learning task (classification or regression), feature importance scores were computed and used to rank features according to their relevance to the model. To quantify the consistency of feature importance rankings between the two feature sets, we calculated the Spearman rank correlation per model and task. Additionally, we evaluated how each model handled colinear features by computing the average SD of importance scores within multi‐feature clusters, divided by the overall SD across all features. Finally, Spearman correlations between ranking outputs were also computed across tasks and models to assess consistency across configurations.

### 5.14 Quantification and visualization of feature effects and interactions

To investigate the contribution and interactions of the analyzed features in our predictive regression models of normalized recombination rate, we employed H‐statistics to quantify interaction strength between features and ALEs plots to visualize both individual and joint effects in a model‐agnostic framework. All computations and visualizations were performed using the iml package in R (Molnar, 2018).

## AUTHOR CONTRIBUTIONS

**Jose A. Montero‐Tena**: Conceptualization; formal analysis; investigation; methodology; software; visualization. **Silvia F. Zanini**: Data curation; investigation; methodology; writing—review and editing. **Gözde Yildiz**: Formal analysis; visualization. **Tobias Kox**: Data curation; investigation. **Amine Abbadi**: Data curation; investigation. **Rod J. Snowdon**: Writing—review and editing. **Agnieszka A. Golicz**: Conceptualization; funding acquisition; methodology; supervision; writing—review and editing

## CONFLICT OF INTEREST STATEMENT

The authors declare no conflicts of interest.

## Supporting information




**Supplementary Figure S1**. Distribution of raw crossover (CO) interval lengths detected with haploMAGIC in two large rapeseed multiparental populations. The x‐axis represents the CO interval size in megabase pairs, and the y‐axis shows the number of crossovers. The vertical solid red line indicates the median, while the vertical dashed red line denotes the mean.
**Supplementary Figure S2**. Distribution of the total number of crossovers per individual. The x‐axis indicates the total number of crossovers per individual across paternal and maternal meiosis and all chromosomes, while the y‐axis shows the number of individuals. The vertical dashed red line marks the filtering threshold of 100 crossovers.
**Supplementary Figure S3**. Distribution of crossover (CO) interval lengths detected with haploMAGIC in two large rapeseed multiparental populations after applying filtering criteria. The x‐axis represents the CO interval size in megabase pairs, and the y‐axis shows the number of crossovers. The vertical solid red line indicates the median, while the vertical dashed red line denotes the mean.
**Supplementary Figure S4**. Genome‐wide recombination rate (in cM/Mbp) by population over 0.3‐Mbp genomic bins, with Pearson correlation coefficients reported for each chromosome. Populations are represented by different colors. Chromosomes are arranged column‐wise by subgenome and row‐wise by chromosome number. Centromere locations are indicated with thick dark grey vertical lines.
**Supplementary Figure S5**. Pairwise relationships among genomic features. The lower triangle displays scatterplots, the upper triangle reports Pearson correlation coefficients (r) with significance (*** P < 0.001, * P < 0.01, * P < 0.05), and the diagonal shows the univariate distributions of each feature. A and C subgenomes, labelled in the diagonal cell corresponding to subgenome, correspond to values 0 and 1 respectively.
**Supplementary Figure S6**. Genome‐wide recombination rate (in cM/Mbp) per SNP marker and feature trends over 2‐Mbp genomic bins. The grey areas represent recombination rate normalized by SNP marker number, with the overlaid colored line showing its value. Features include CpG methylation rate, transposable element (TE) fraction, gene fraction, gene expression level ($\mathrm{lo}g_{10}$ TPM + 0.1), and SNP content, each normalized by their genome‐wide maximum and represented by light blue, purple, red, pink, and green lines, respectively. Chromosomes are arranged column‐wise by subgenome and row‐wise by chromosome number. Centromere locations are indicated with thick dark grey vertical lines.
**Supplementary Figure S7**. Feature significance ranking based on $\mathrm{lo}g_{10}$ P‐values from two‐sample statistical tests. The C subgenome values were obtained using the chi‐square test, whereas all other values were derived from the t‐test. The vertical dashed line marks the significance threshold (P = 0.1). Red bars represent features positively related with recombination (enriched in hotspots), whereas blue bars represent features negatively related with recombination (depleted in hotspots).
**Supplementary Figure S8**. Chromosome‐wise mean number of crossovers detected by population. The x‐axis represents generation, the y‐axis shows the mean total number of crossovers, and colors indicate different populations.
**Supplementary Figure S9**. Chromosome‐wise SNP density in number of SNPs per megabase pair (Mbp). The x‐axis shows chromosome names, and the y‐axis shows SNP density per Mbp. Light green bars represent the A subgenome, and dark green bars represent the C subgenome. Density values are indicated above each bar. Additional information, including total SNP counts and mean chromosome size per subgenome, is provided in the upper right corner of the figure.
**Supplementary Figure S10**. Probability density (left) and distribution across chromosome arms (right) in the A (orange) and C (blue) subgenomes of several features, plotted from top to bottom: retrotransposon coverage; CpG methylation, CHG methylation, CHH methylation, and TE body CHH methylation; gene expression; gene coverage; and recombination rate. Each row contains two plots corresponding to the feature indicated on the left. For each feature, both plots were derived from the same set of 0.3‐Mbp bins. In the left panels, the density curves represent each subgenome separately (A = orange, C = blue), and the Y axis shows the kernel density estimate of the feature values represented in the X axis. In the right panels, the Y axis shows the feature values, and the X axis shows the relative position along the chromosome arm (Distance from telomere’; 0 = telomere, 1 = centromere). LOESS regression lines were fitted to the A (orange) and C (blue) subgenome values.
**Supplementary Figure S11**. Spearman rank correlation in feature importance rankings between classification and regression tasks for four algorithms when all features were present (blue) and when only the best representative per cluster was included (red).
**Supplementary Figure S12**. Heatmap of Spearman correlation coefficients in feature importance rankings across the four machine learning algorithms used in the analysis.
**Supplementary Figure S13**. Receiver operating characteristic (ROC) curve of the random forest classifier developed using genome‐wide data for hotspot prediction. The curve was constructed using the original feature values and estimated probabilities. The area under the ROC curve (AUROC) is annotated.
**Supplementary Figure S14**. Feature rankings by decreasing importance scores obtained from each regression model (top left: boosted trees; top right: decision tree; bottom left: linear regression; bottom right: random forest) predicting recombination rates with genome‐wide data. For the random forest, boosted trees and decision tree models, features positively correlated with predicted recombination rates are shown in red, while negatively correlated features are shown in blue. For the logistic regression model, colors correspond to the sign of the coefficients of terms assigned by the model.
**Supplementary Figure S15**. Feature ranking by decreasing Gini impurity‐based importance scores obtained from the random forest classifier in the hotspot prediction task using genome‐wide data. Features that are more abundant in the hotspots are shown in red, while those more abundant in the non‐hotspots are shown in blue.
**Supplementary Figure S16**. Feature rankings by decreasing importance scores obtained from each classifier (top left: random forest; top right: boosted trees; bottom left: logistic regression; bottom right: decision tree) predicting hotspot location with genome‐wide data. For the random forest, boosted trees and decision tree models, features that are more abundant in the hotspots are shown in red, while those more abundant in the non‐hotspots are shown in blue. For the logistic regression model, colors correspond to the sign of the coefficients of terms assigned by the model.
**Supplementary Figure S17**. Features ranked by decreasing interaction strength, as measured by Friedman's H‐statistics.
**Supplementary Figure S18**. Feature ranking by decreasing Gini impurity‐based importance scores obtained by the random forest regression models predicting recombination rates with A‐subgenome feature data. Features positively correlated with recombination are in red, whereas the negatively correlated ones are in blue.
**Supplementary Figure S19**. Feature ranking by decreasing Gini impurity‐based importance scores obtained by the random forest regression models predicting recombination rates with C‐subgenome feature data. Features positively correlated with recombination are in red, whereas the negatively correlated ones are in blue.
**Supplementary Figure S20**. Accumulated local effect (ALE) curves for features in the model built using A‐subgenome data. The x‐axis shows feature values, and the y‐axis shows the ALE, centered around zero.
**Supplementary Figure S21**. Accumulated local effect (ALE) curves for features in the model built using C‐subgenome data. The x‐axis shows feature values, and the y‐axis shows the ALE, centered around zero.
**Supplementary Figure S22**. Number of crossovers detected with haploMAGIC under the min2/5/3 parameter setting, separated by generation.
**Supplementary Figure S23**. Line plots show the evolution of single‐base and weighted methylation rates in the CHH context across 0.3‐Mbp genomic bins for each chromosome. Blue lines represent single‐base methylation rate value, and orange lines represent weighted methylation rate values. Values were obtained with the formulas reported in (Schultz et al., 2012) and constitute raw average rates per bin, previous to the final smoothed values used in machine learning tasks. Pearson's correlation coefficient (r) between the values per chromosome is indicated in the top right corner of each chromosome.

## Data Availability

Data generated in this study has been made available at https://osf.io/362d8/. The R code used to reproduce the genome‐wide, A subgenome, and C subgenome analyses is available at https://jamonterotena.github.io/bnapus.reco.ml/.

## References

[tpg270209-bib-0001] Abdollahi, N. , Herzog, E. , Abbadi, A. , Snowdon, R. J. , & Golicz, A. A. (2024). Analysis of the winter oilseed rape recombination landscape suggests maternal–paternal bias. Genome, 67, 481–497. 10.1139/gen-2023-0110 39431738

[tpg270209-bib-0002] Albrechtsen, A. , Nielsen, F. C. , & Nielsen, R. (2010). Ascertainment biases in SNP chips affect measures of population divergence. Molecular Biology and Evolution, 27(11), 2534–2547. 20558595 10.1093/molbev/msq148 PMC3107607

[tpg270209-bib-0003] Allender, C. J. , & King, G. J. (2010). Origins of the amphiploid species *Brassica napus* L. investigated by chloroplast and nuclear molecular markers. BMC Plant Biology, 10(1), Article 54. 20350303 10.1186/1471-2229-10-54 PMC2923528

[tpg270209-bib-0004] Bernstein, H. , & Bernstein, C. (2010). Evolutionary origin of meiosis. Bioscience, 60(7), 498–505.

[tpg270209-bib-0005] Boideau, F. , Richard, G. , Coriton, O. , Huteau, V. , Belser, C. , Deniot, G. , Eber, F. , Falentin, C. , Ferreira De Carvalho, J. , Gilet, M. , Lodé‐Taburel, M. , Maillet, L. , Morice, J. , Trotoux, G. , Aury, J.‐M. , Chèvre, A.‐M. , & Rousseau‐Gueutin, M. (2022). Epigenomic and structural events preclude recombination in *Brassica napus* . New Phytologist, 234(2), 545–559. 35092024 10.1111/nph.18004

[tpg270209-bib-0006] Bray, N. L. , Pimentel, H. , Melsted, P. , & Pachter, L. (2016). Near‐optimal probabilistic RNA‐seq quantification. Nature Biotechnology, 34(5), 525–527. 10.1038/nbt.3519 27043002

[tpg270209-bib-0007] Christophorou, N. , She, W. , Long, J. , Hurel, A. , Beaubiat, S. , Idir, Y. , Tagliaro‐Jahns, M. , Chambon, A. , Solier, V. , Vezon, D. , Grelon, M. , Feng, X. , Bouché, N. , & Mézard, C. (2020). AXR1 affects DNA methylation independently of its role in regulating meiotic crossover localization. PLoS Genetics, 16(6), Article e1008894. 32598340 10.1371/journal.pgen.1008894 PMC7351236

[tpg270209-bib-0008] Clarke, W. E. , Higgins, E. E. , Plieske, J. , Wieseke, R. , Sidebottom, C. , Khedikar, Y. , Batley, J. , Edwards, D. , Meng, J. , Li, R. , Lawley, C. T. , Pauquet, J. , Laga, B. , Cheung, W. , Iniguez‐Luy, F. , Dyrszka, E. , Rae, S. , Stich, B. , Snowdon, R. J. , … Parkin, I. A. P. (2016). A high‐density SNP genotyping array for *Brassica napus* and its ancestral diploid species based on optimised selection of single‐locus markers in the allotetraploid genome. Theoretical and Applied Genetics, 129(10), 1887–1899. 27364915 10.1007/s00122-016-2746-7 PMC5025514

[tpg270209-bib-0009] Cutter, A. D. , & Payseur, B. A. (2013). Genomic signatures of selection at linked sites: Unifying the disparity among species. Nature Reviews Genetics, 14(4), 262–274. 10.1038/nrg3425 PMC4066956 23478346

[tpg270209-bib-0010] Demirci, S. , Peters, S. A. , De Ridder, D. , & Van Dijk, A. D. J. (2018). DNA sequence and shape are predictive for meiotic crossovers throughout the plant kingdom. The Plant Journal, 95(4), 686–699. 10.1111/tpj.13979 29808512

[tpg270209-bib-0011] Demirci, S. , van Dijk, A. D. , Sanchez Perez, G. , Aflitos, S. A. , de Ridder, D. , & Peters, S. A. (2017). Distribution, position and genomic characteristics of crossovers in tomato recombinant inbred lines derived from an interspecific cross between *Solanum lycopersicum* and *Solanum pimpinellifolium* . The Plant Journal, 89(3), 554–564. 27797425 10.1111/tpj.13406

[tpg270209-bib-0012] Friedman, J. H. , & Popescu, B. E. (2008). Predictive learning via rule ensembles. The Annals of Applied Statistics, 2(3), 916–954.

[tpg270209-bib-0013] Gent, J. I. , Ellis, N. A. , Guo, L. , Harkess, A. E. , Yao, Y. , Zhang, X. , & Dawe, R. K. (2013). CHH islands: De novo DNA methylation in near‐gene chromatin regulation in maize. Genome Research, 23(4), 628–637. 23269663 10.1101/gr.146985.112 PMC3613580

[tpg270209-bib-0014] Grandbastien, M. (2015). LTR retrotransposons, handy hitchhikers of plant regulation and stress response. Biochimica et Biophysica Acta (BBA)—Gene Regulatory Mechanisms, 1849(4), 403–416. 25086340 10.1016/j.bbagrm.2014.07.017

[tpg270209-bib-0015] Higgins, E. E. , Clarke, W. E. , Howell, E. C. , Armstrong, S. J. , & Parkin, I. A. P. (2018). Detecting de novo homoeologous recombination events in cultivated *Brassica napus* using a genome‐wide SNP array. G3: Genes, Genomes, Genetics, 8(8), 2673–2683. 29907649 10.1534/g3.118.200118 PMC6071606

[tpg270209-bib-0016] Hsu, Y.‐M. , Falque, M. , & Martin, O. C. (2022). Quantitative modelling of fine‐scale variations in the *Arabidopsis thaliana* crossover landscape. Quantitative Plant Biology, 3, Article e3. 37077963 10.1017/qpb.2021.17 PMC10095869

[tpg270209-bib-0017] Hyndman, R. J. , & Khandakar, Y. (2008). Automatic time series forecasting: The forecast package for R. Journal of Statistical Software, 27(3), 1–22.

[tpg270209-bib-0018] Krenzer, D. , Frisch, M. , Beckmann, K. , Kox, T. , Flachenecker, C. , Abbadi, A. , Snowdon, R. , & Herzog, E. (2024). Simulation‐based establishment of base pools for a hybrid breeding program in winter rapeseed. Theoretical and Applied Genetics, 137(1), Article 16. 38189816 10.1007/s00122-023-04519-3 PMC10774156

[tpg270209-bib-0019] Krueger, F. , & Andrews, S. R. (2011). Bismark: A flexible aligner and methylation caller for bisulfite‐seq applications. Bioinformatics, 27(11), 1571–1572. 21493656 10.1093/bioinformatics/btr167 PMC3102221

[tpg270209-bib-0020] Kuhn, M. , & Wickham, H. (2020). Tidymodels: A collection of packages for modeling and machine learning using tidyverse principles . https://www.tidymodels.org/

[tpg270209-bib-0021] Lambing, C. , Franklin, F. C. H. , & Wang, C.‐J. R. (2017). Understanding and manipulating meiotic recombination in plants. Plant Physiology, 173(3), 1530–1542. 28108697 10.1104/pp.16.01530 PMC5338670

[tpg270209-bib-0022] Law, J. A. , & Jacobsen, S. E. (2010). Establishing, maintaining and modifying DNA methylation patterns in plants and animals. Nature Reviews Genetics, 11(3), 204–220. 10.1038/nrg2719 PMC3034103 20142834

[tpg270209-bib-0023] Lee, H. , Chawla, H. S. , Obermeier, C. , Dreyer, F. , Abbadi, A. , & Snowdon, R. (2020). Chromosome‐scale assembly of winter oilseed rape *Brassica napus* . Frontiers in Plant Science, 11, Article 496. 32411167 10.3389/fpls.2020.00496 PMC7202327

[tpg270209-bib-0024] Lukens, L. N. , Pires, J. C. , Leon, E. , Vogelzang, R. , Oslach, L. , & Osborn, T. (2006). Patterns of sequence loss and cytosine methylation within a population of newly resynthesized *Brassica napus* allopolyploids. Plant Physiology, 140(1), 336–348. 16377753 10.1104/pp.105.066308 PMC1326055

[tpg270209-bib-0025] Marçais, G. , Delcher, A. L. , Phillippy, A. M. , Coston, R. , Salzberg, S. L. , & Zimin, A. (2018). MUMmer4: A fast and versatile genome alignment system. PLoS Computational Biology, 14, Article e1005944. 29373581 10.1371/journal.pcbi.1005944 PMC5802927

[tpg270209-bib-0026] Martin, G. T. , Seymour, D. K. , & Gaut, B. S. (2021). CHH methylation islands: A nonconserved feature of grass genomes that is positively associated with transposable elements but negatively associated with gene‑body methylation. Genome Biology and Evolution, 13(8), Article evab144. 34146109 10.1093/gbe/evab144 PMC8374106

[tpg270209-bib-0027] Mason, A. S. , Rousseau‐Gueutin, M. , Morice, J. , Bayer, P. E. , Besharat, N. , Cousin, A. , Pradhan, A. , Parkin, I. A. P. , Chèvre, A.‐M. , Batley, J. , & Nelson, M. N. (2016). Centromere locations in Brassica A and C genomes revealed through half‑tetrad analysis. Genetics, 202(2), 513–523. 26614742 10.1534/genetics.115.183210 PMC4788232

[tpg270209-bib-0028] Molnar, C. (2018). Interpretable machine learning: A guide for making black box models explainable. Leanpub.

[tpg270209-bib-0029] Montero‐Tena, J. A. , Abdollahi‐Sisi, N. , Kox, T. , Abbadi, A. , Snowdon, R. J. , & Golicz, A. A. (2024). haploMAGIC: Accurate phasing and detection of recombination in multiparental populations despite genotyping errors. G3: Genes, Genomes, Genetics, 14(8), Article jkae109. 38808682 10.1093/g3journal/jkae109 PMC11304941

[tpg270209-bib-0030] Peñuela, M. , Finke, J. , & Rocha, C. (2024). Methylomes as key features for predicting recombination in some plant species. Plant Molecular Biology, 114, Article 25. 38457042 10.1007/s11103-023-01396-8 PMC10924001

[tpg270209-bib-0031] Peñuela, M. , Gallo‐Franco, J. J. , Finke, J. , Rocha, C. , Gkanogiannis, A. , Ghneim‐Herrera, T. , & Lorieux, M. (2022). Methylation in the CHH context allows to predict recombination in rice. International Journal of Molecular Sciences, 23, Article 12505. 36293364 10.3390/ijms232012505 PMC9604423

[tpg270209-bib-0032] Qian, L. , Qian, W. , & Snowdon, R. J. (2014). Sub‐genomic selection patterns as a signature of breeding in the allopolyploid *Brassica napus* genome. BMC Genomics, 15(1), Article 1170. 25539568 10.1186/1471-2164-15-1170 PMC4367848

[tpg270209-bib-0033] Quinlan, A. R. , & Hall, I. M. (2010). BEDTools: A flexible suite of utilities for comparing genomic features. Bioinformatics, 26(6), 841–842. 20110278 10.1093/bioinformatics/btq033 PMC2832824

[tpg270209-bib-0034] R Core Team . (2024). R: A language and environment for statistical computing . R Foundation for Statistical Computing.

[tpg270209-bib-0035] Rodgers‐Melnick, E. , Bradbury, P. J. , Elshire, R. J. , Glaubitz, J. C. , Acharya, C. B. , Mitchell, S. E. , Li, C. , Li, Y. , & Buckler, E. S. (2015). Recombination in diverse maize is stable, predictable, and associated with genetic load. Proceedings of the National Academy of Sciences of the USA, 112(12), 3823–3828. 25775595 10.1073/pnas.1413864112 PMC4378432

[tpg270209-bib-0036] Rousseau‐Gueutin, M. , Belser, C. , Da Silva, C. , Richard, G. , Istace, B. , Cruaud, C. , Falentin, C. , Boideau, F. , Boutte, J. , Delourme, R. , Deniot, G. , Engelen, S. , De Carvalho, J. F. , Lemainque, A. , Maillet, L. , Morice, J. , Wincker, P. , Denoeud, F. , Chèvre, A.‐M. , & Aury, J.‐M. (2020). Long‐read assembly of the *Brassica napus* reference genome Darmor‐bzh. GigaScience, 9(12), Article giaa137. 33319912 10.1093/gigascience/giaa137 PMC7736779

[tpg270209-bib-0037] Salomé, P. A. , Bomblies, K. , Fitz, J. , Laitinen, R. A. E. , Warthmann, N. , Yant, L. , & Weigel, D. (2012). The recombination landscape in *Arabidopsis thaliana* F_2_ populations. Heredity, 108, 447–455. 22072068 10.1038/hdy.2011.95 PMC3313057

[tpg270209-bib-0038] Schultz, M. D. , Schmitz, R. J. , & Ecker, J. R. (2012). ‘Leveling’ the playing field for analyses of single‐base resolution DNA methylomes. Trends in Genetics, 28(12), 583–585. 23131467 10.1016/j.tig.2012.10.012 PMC3523709

[tpg270209-bib-0039] Scott, M. F. , Ladejobi, O. , Amer, S. , Bentley, A. R. , Biernaskie, J. , Boden, S. A. , Clark, M. , Dell'Acqua, M. , Dixon, L. E. , Filippi, C. V. , Fradgley, N. , Gardner, K. A. , Mackay, I. J. , O'Sullivan, D. , Percival‐Alwyn, L. , Roorkiwal, M. , Singh, R. K. , Thudi, M. , Varshney, R. K. , … Mott, R. (2020). Multi‐parent populations in crops: A toolbox integrating genomics and genetic mapping with breeding. Heredity, 125(6), 396–416. 32616877 10.1038/s41437-020-0336-6 PMC7784848

[tpg270209-bib-0040] Si, W. , Yuan, Y. , Huang, J. , Zhang, X. , Zhang, Y. , Zhang, Y. , Tian, D. , Wang, C. , Yang, Y. , & Yang, S. (2015). Widely distributed hot and cold spots in meiotic recombination as shown by the sequencing of rice F_2_ plants. New Phytologist, 206(4), 1491–1502. 25664766 10.1111/nph.13319

[tpg270209-bib-0041] Smit, A. F. A. , Hubley, R. , & Green, P. (2013–2023). RepeatMasker open‐4.0 . https://www.repeatmasker.org/

[tpg270209-bib-0042] Song, Q. , & Chen, Z. J. (2015). Epigenetic and developmental regulation in plant polyploids. Current Opinion in Plant Biology, 24, 101–109. 25765928 10.1016/j.pbi.2015.02.007 PMC4395545

[tpg270209-bib-0043] Stroud, H. , Do, T. , Du, J. , Zhong, X. , Feng, S. , Johnson, L. , Patel, D. J. , & Jacobsen, S. E. (2014). Non‐CG methylation patterns shape the epigenetic landscape in *Arabidopsis* . Nature Structural & Molecular Biology, 21(1), 64–72. 10.1038/nsmb.2735 PMC4103798 24336224

[tpg270209-bib-0044] Stroud, H. , Hale, C. J. , Feng, S. , Caro, E. , Jacob, Y. , Michaels, S. D. , & Jacobsen, S. E. (2012). DNA methyltransferases are required to induce heterochromatic re‐replication in *Arabidopsis* . PLoS Genetics, 8(7), Article e1002808. 22792077 10.1371/journal.pgen.1002808 PMC3390372

[tpg270209-bib-0045] Su, W. , Ou, S. , Hufford, M. B. , & Peterson, T. (2021). A tutorial of EDTA: Extensive de novo TE annotator. Methods in Molecular Biology, 2250, 55–67. 33900591 10.1007/978-1-0716-1134-0_4

[tpg270209-bib-0046] Vincenten, N. , Kuhl, L.‐M. , Lam, I. , Oke, A. , Kerr, A. R. W. , Hochwagen, A. , Fung, J. , Keeney, S. , Vader, G. , & Marston, A. L. (2015). The kinetochore prevents centromere‐proximal crossover recombination during meiosis. Elife, 4, Article e10850. 26653857 10.7554/eLife.10850 PMC4749563

[tpg270209-bib-0047] Wang, M. , King, G. J. , Shi, L. , Li, R. , Zhang, Y. , Wang, X. , Meng, J. , Tu, J. , & Zou, J. (2023). Genome‐wide recombination variation in biparental segregating and reciprocal backcross populations provides information for introgression breeding in *Brassica napus* . The Crop Journal, 11(1), 208–219.

[tpg270209-bib-0048] Wang, Z. , Wu, X. , Wu, Z. , An, H. , Yi, B. , Wen, J. , Ma, C. , Shen, J. , Fu, T. , & Tu, J. (2018). Genome‑wide DNA methylation comparison between *Brassica napus* genic male sterile line and restorer line. International Journal of Molecular Sciences, 19(9), Article 2689. 30201884 10.3390/ijms19092689 PMC6165103

[tpg270209-bib-0049] Wei, L. , Xiao, M. , An, Z. , Ma, B. , Mason, A. S. , Qian, W. , Li, J. , & Fu, D. (2013). New insights into nested long terminal repeat retrotransposons in *Brassica* species. Molecular Plant, 6(2), 470–482. 22930733 10.1093/mp/sss081

[tpg270209-bib-0050] Wijnker, E. , & Dejong, H. (2008). Managing meiotic recombination in plant breeding. Trends in Plant Science, 13(12), 640–646. 18948054 10.1016/j.tplants.2008.09.004

[tpg270209-bib-0051] Wijnker, E. , Velikkakam James, G. , Ding, J. , Becker, F. , Klasen, J. R. , Rawat, V. , Rowan, B. A. , De Jong, D. F. , De Snoo, C. B. , Zapata, L. , Huettel, B. , De Jong, H. , Ossowski, S. , Weigel, D. , Koornneef, M. , Keurentjes, J. J. B. , & Schneeberger, K. (2013). The genomic landscape of meiotic crossovers and gene conversions in *Arabidopsis thaliana* . Elife, 2, Article e01426. 24347547 10.7554/eLife.01426 PMC3865688

[tpg270209-bib-0052] Yan, S. , He, J. , Tang, M. , Ming, B. , Li, H. , Fan, S. , Xiong, Y. , Chao, H. , Zhang, L. , Wang, A. , & Li, M. (2023). Dissecting the meiotic recombination patterns in a *Brassica napus* double haploid population using 60K SNP array. International Journal of Molecular Sciences, 24, Article 4469. 36901901 10.3390/ijms24054469 PMC10003086

[tpg270209-bib-0053] Yang, S. , Wang, L. , Huang, J. , Zhang, X. , Yuan, Y. , Chen, J.‐Q. , Hurst, L. D. , & Tian, D. (2015). Parent–progeny sequencing indicates higher mutation rates in heterozygotes. Nature, 523(7561), 463–467. 26176923 10.1038/nature14649

[tpg270209-bib-0054] Yelina, N. E. , Choi, K. , Chelysheva, L. , Macaulay, M. , De Snoo, B. , Wijnker, E. , Miller, N. , Drouaud, J. , Grelon, M. , Copenhaver, G. P. , Mezard, C. , Kelly, K. A. , & Henderson, I. R. (2012). Epigenetic remodeling of meiotic crossover frequency in *Arabidopsis thaliana* DNA methyltransferase mutants. PLoS Genetics, 8(8), Article e1002844. 22876192 10.1371/journal.pgen.1002844 PMC3410864

[tpg270209-bib-0055] Yelina, N. E. , Lambing, C. , Hardcastle, T. J. , Zhao, X. , Santos, B. , & Henderson, I. R. (2015). DNA methylation epigenetically silences crossover hot spots and controls chromosomal domains of meiotic recombination in *Arabidopsis* . Genes & Development, 29(20), 2183–2202. 26494791 10.1101/gad.270876.115 PMC4617981

[tpg270209-bib-0056] Zanini, S. F. , Rockenbach, K. , Nguyen, A. , Arslan, K. , Yildiz, G. , Snowdon, R. J. , & Golicz, A. A. (2025). Integrative epigenomic analysis uncovers asymmetry of enhancer activity in *Brassica napus* . *bioRxiv*. 10.1101/2025.10.31.685802

[tpg270209-bib-0057] Zhang, X. (2008). The epigenetic landscape of plants. Science, 320(5875), 489–492. 18436779 10.1126/science.1153996

[tpg270209-bib-0058] Zhang, Q. , Guan, P. , Zhao, L. , Ma, M. , Xie, L. , Li, Y. , Zheng, R. , Ouyang, W. , Wang, S. , Li, H. , Zhang, Y. , Peng, Y. , Cao, Z. , Zhang, W. , Xiao, Q. , Xiao, Y. , Fu, T. , Li, G. , Li, X. , & Shen, J. (2021). Asymmetric epigenome maps of subgenomes reveal imbalanced transcription and distinct evolutionary trends in *Brassica napus* . Molecular Plant, 14(4), 604–619. 33387675 10.1016/j.molp.2020.12.020

[tpg270209-bib-0059] Zhao, M. , Ku, J.‐C. , Liu, B. , Yang, D. , Yin, L. , Ferrell, T. J. , Stoll, C. E. , Guo, W. , Zhang, X. , Wang, D. , Wang, C. J. R. , & Lisch, D. (2021). The mop1 mutation affects the recombination landscape in maize. Proceedings of the National Academy of Sciences of the USA, 118(7), Article e2009475118. 33558228 10.1073/pnas.2009475118 PMC7896300

