# Supplementary material for: Machine learning and multi‐omic analysis reveal contrasting recombination landscape of A and C subgenomes of winter oilseed rape
Source: Plant Genome. 2026 Mar 19;19(1):e70209. doi: 10.1002/tpg2.70209 (PMC13003170; doi:10.1002/tpg2.70209)
Supplement: Supplementary file 1 — Supplementary Figure S1. Distribution of raw crossover (CO) interval lengths detected with haploMAGIC in two large rapeseed multiparental populations. The x‐axis represents the CO interval size in megabase pairs, and the y‐axis shows the number of crossovers. The vertical solid red line indicates the median, while the vertical dashed red line denotes the mean. Supplementary Figure S2. Distribution of the total number of crossovers per individual. The x‐axis indicates the total number of crossovers per individual across paternal and maternal meiosis and all chromosomes, while the y‐axis shows the number of individuals. The vertical dashed red line marks the filtering threshold of 100 crossovers. Supplementary Figure S3. Distribution of crossover (CO) interval lengths detected with haploMAGIC in two large rapeseed multiparental populations after applying filtering criteria. The x‐axis represents the CO interval size in megabase pairs, and the y‐axis shows the number of crossovers. The vertical solid red line indicates the median, while the vertical dashed red line denotes the mean. Supplementary Figure S4. Genome‐wide recombination rate (in cM/Mbp) by population over 0.3‐Mbp genomic bins, with Pearson correlation coefficients reported for each chromosome. Populations are represented by different colors. Chromosomes are arranged column‐wise by subgenome and row‐wise by chromosome number. Centromere locations are indicated with thick dark grey vertical lines. Supplementary Figure S5. Pairwise relationships among genomic features. The lower triangle displays scatterplots, the upper triangle reports Pearson correlation coefficients (r) with significance (*** P < 0.001, * P < 0.01, * P < 0.05), and the diagonal shows the univariate distributions of each feature. A and C subgenomes, labelled in the diagonal cell corresponding to subgenome, correspond to values 0 and 1 respectively. Supplementary Figure S6. Genome‐wide recombination rate (in cM/Mbp) per SNP marker and featur [file TPG2-19-e70209-s001.pdf]

# Index of Supplementary Figures


- Supplementary Figure S1..........................................P2
- Supplementary Figure S2..........................................P3
- Supplementary Figure S3..........................................P4
- Supplementary Figure S4..........................................P5
- Supplementary Figure S5..........................................P6
- Supplementary Figure S6..........................................P7
- Supplementary Figure S7..........................................P8
- Supplementary Figure S8..........................................P9
- Supplementary Figure S9..........................................P10
- Supplementary Figure S10........................................P11
- Supplementary Figure S11........................................P12
- Supplementary Figure S12........................................P13
- Supplementary Figure S13........................................P14
- Supplementary Figure S14........................................P15
- Supplementary Figure S15........................................P16
- Supplementary Figure S16........................................P17
- Supplementary Figure S17........................................P18
- Supplementary Figure S18........................................P19
- Supplementary Figure S19........................................P20
- Supplementary Figure S20........................................P21
- Supplementary Figure S21........................................P22
- Supplementary Figure S22........................................P23
- Supplementary Figure S23........................................P24

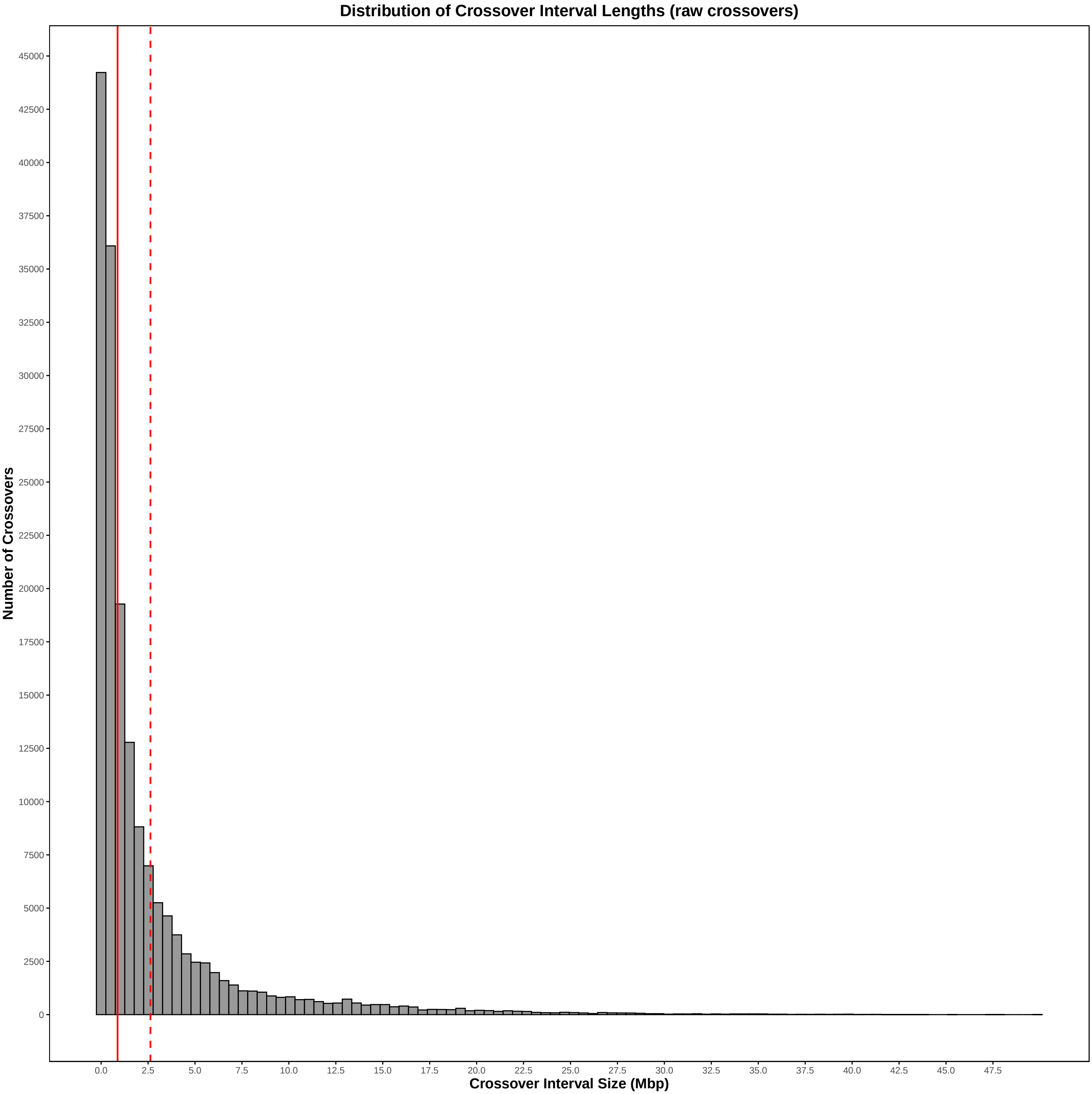

Distribution of Crossover Interval Lengths (raw crossovers)
Number of Crossovers
45000
42500
40000
37500
35000
32500
30000
27500
25000
22500
20000
17500
15000
12500
10000
7500
5000
2500
0
0.0
2.5
5.0
7.5
10.0
12.5
15.0
17.5
20.0
22.5
25.0
27.5
30.0
32.5
35.0
37.5
40.0
42.5
45.0
47.5
Crossover Interval Size (Mbp)

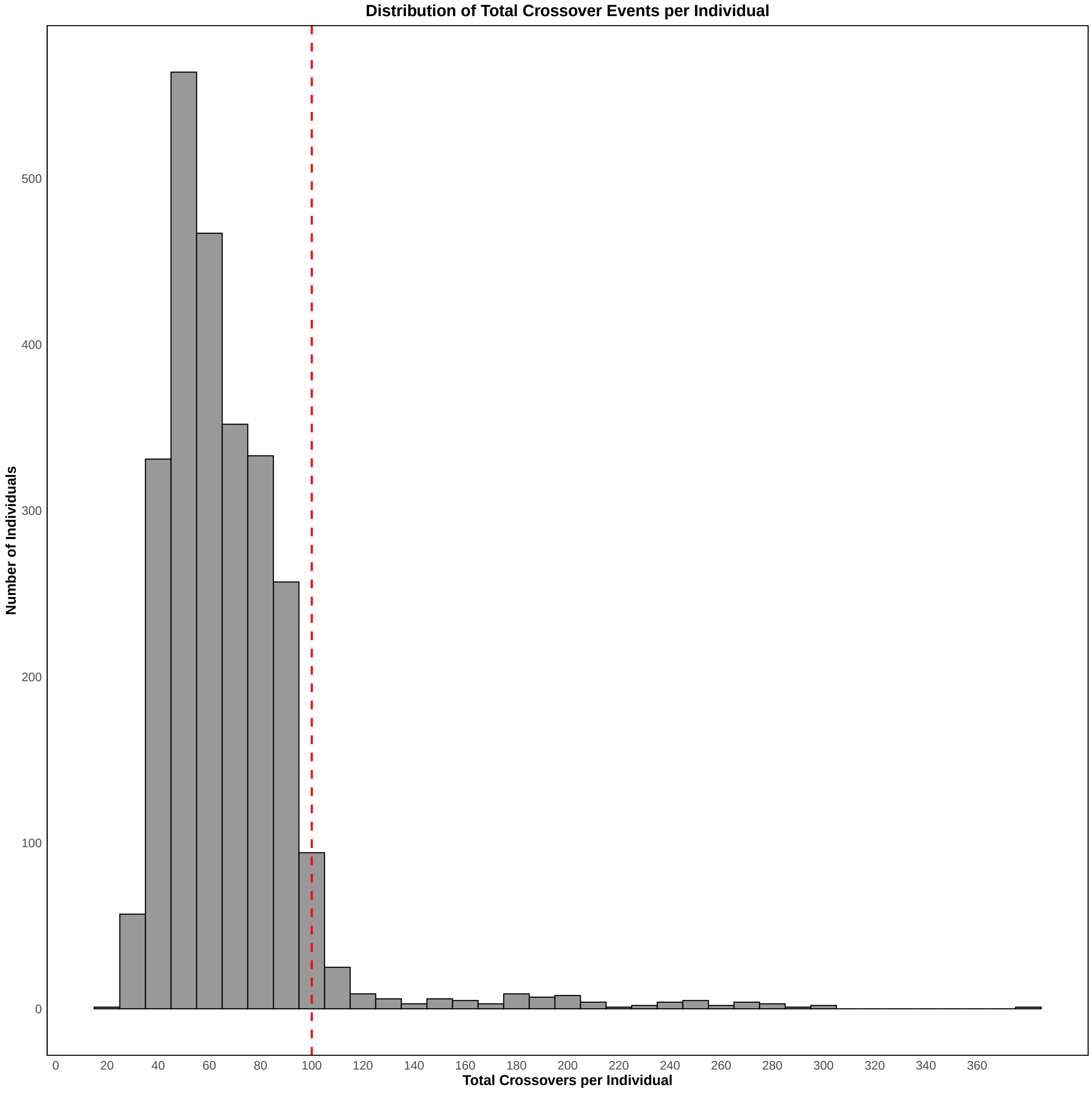

Distribution of Total Crossover Events per Individual
Number of Individuals
500
400
300
200
100
0
0
20
40
60
80
100
120
140
160
180
200
220
240
260
280
300
320
340
360
Total Crossovers per Individual

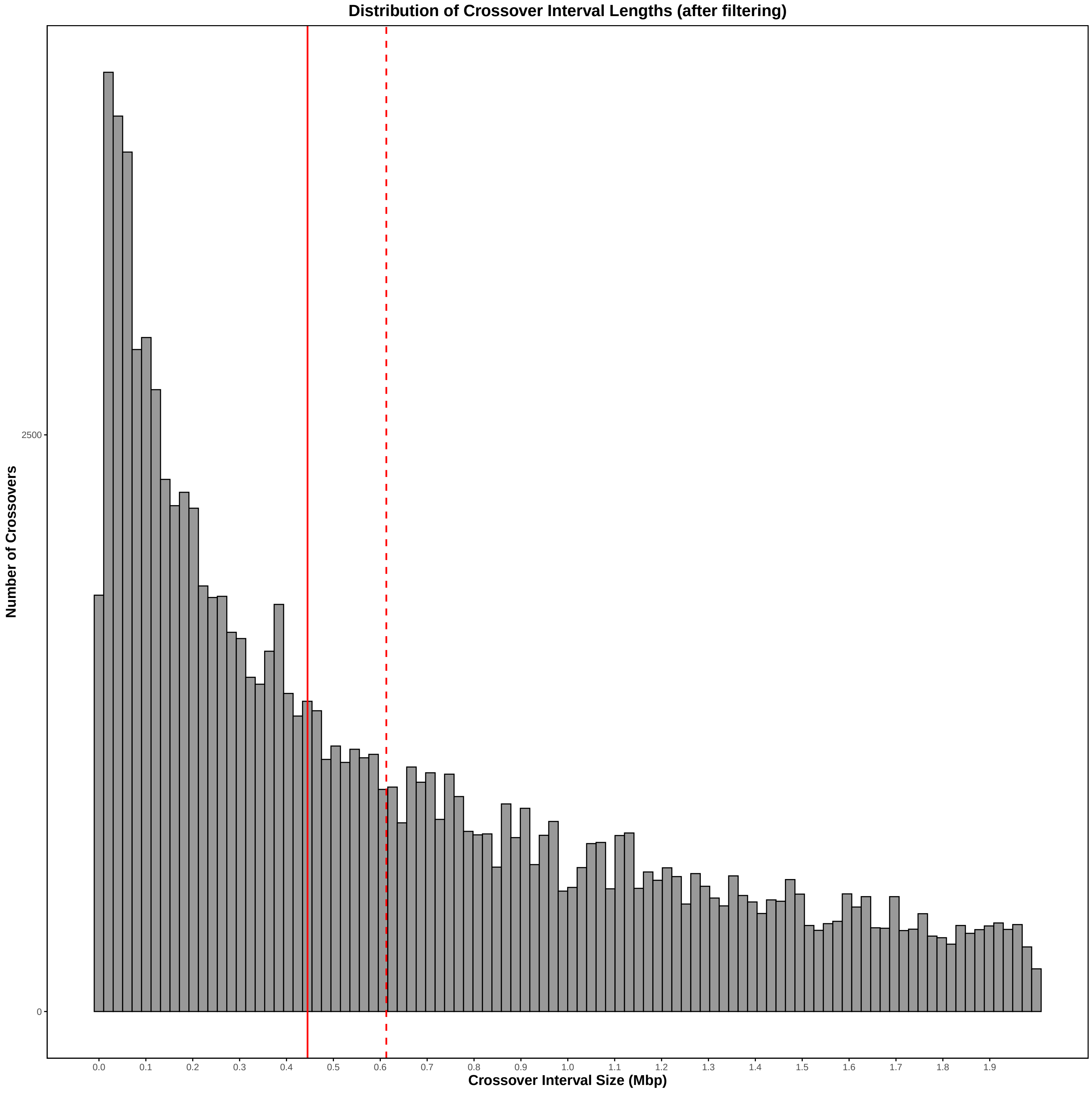
Distribution of Crossover Interval Lengths (after filtering)
Number of Crossovers
2500
0
0.0
0.1
0.2
0.3
0.4
0.5
0.6
0.7
0.8
0.9
1.0
1.1
1.2
1.3
1.4
1.5
1.6
1.7
1.8
1.9
Crossover Interval Size (Mbp)

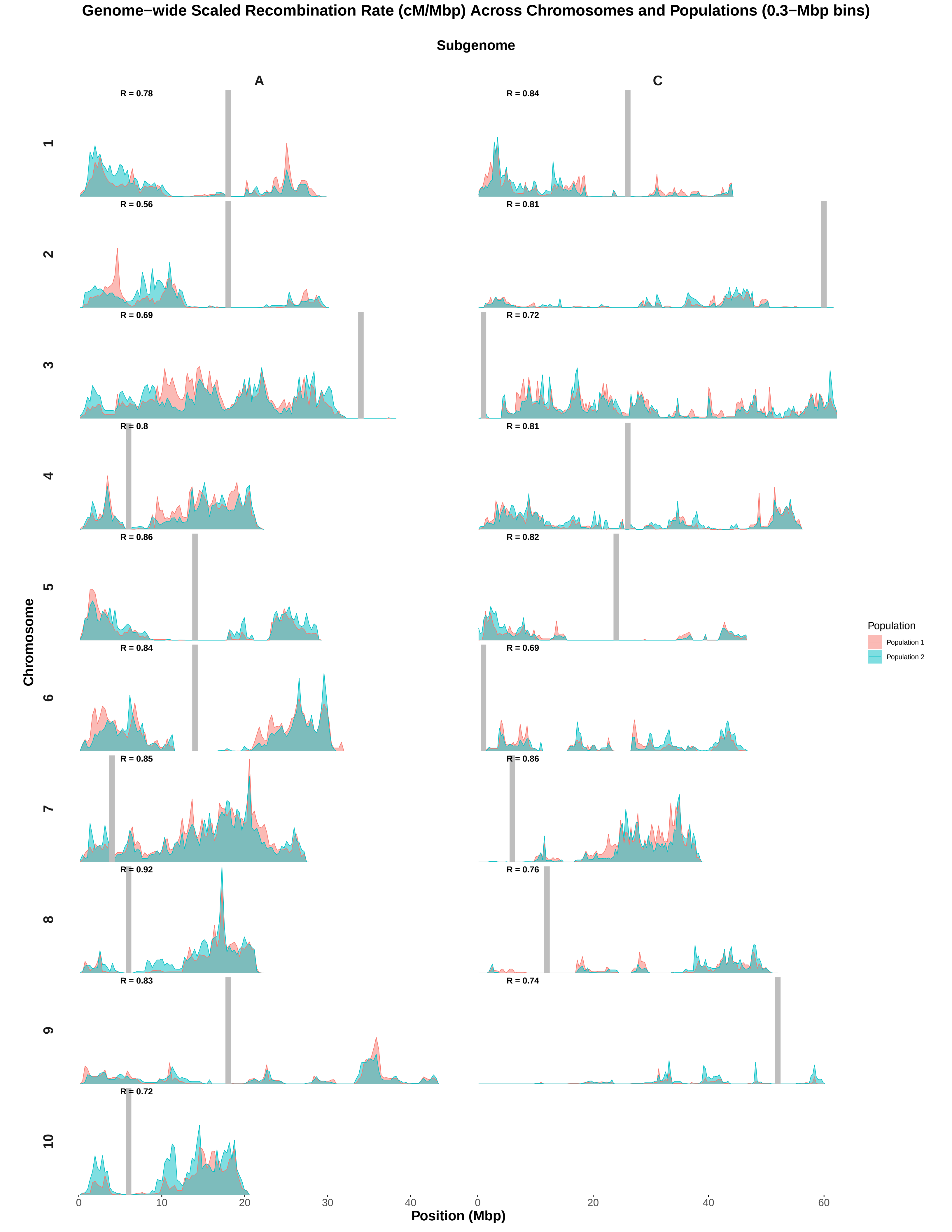

Genome−wide Scaled Recombination Rate (cM/Mbp) Across Chromosomes and Populations (0.3−Mbp bins)
Subgenome
A
C
Chromosome
1
2
3
4
5
6
7
8
9
10
R = 0.78
R = 0.84
R = 0.56
R = 0.81
R = 0.69
R = 0.72
R = 0.8
R = 0.81
R = 0.86
R = 0.82
R = 0.84
R = 0.69
R = 0.85
R = 0.86
R = 0.92
R = 0.76
R = 0.83
R = 0.74
R = 0.72
Position (Mbp)
0
10
20
30
40
0
20
40
60
Population
Population 1
Population 2

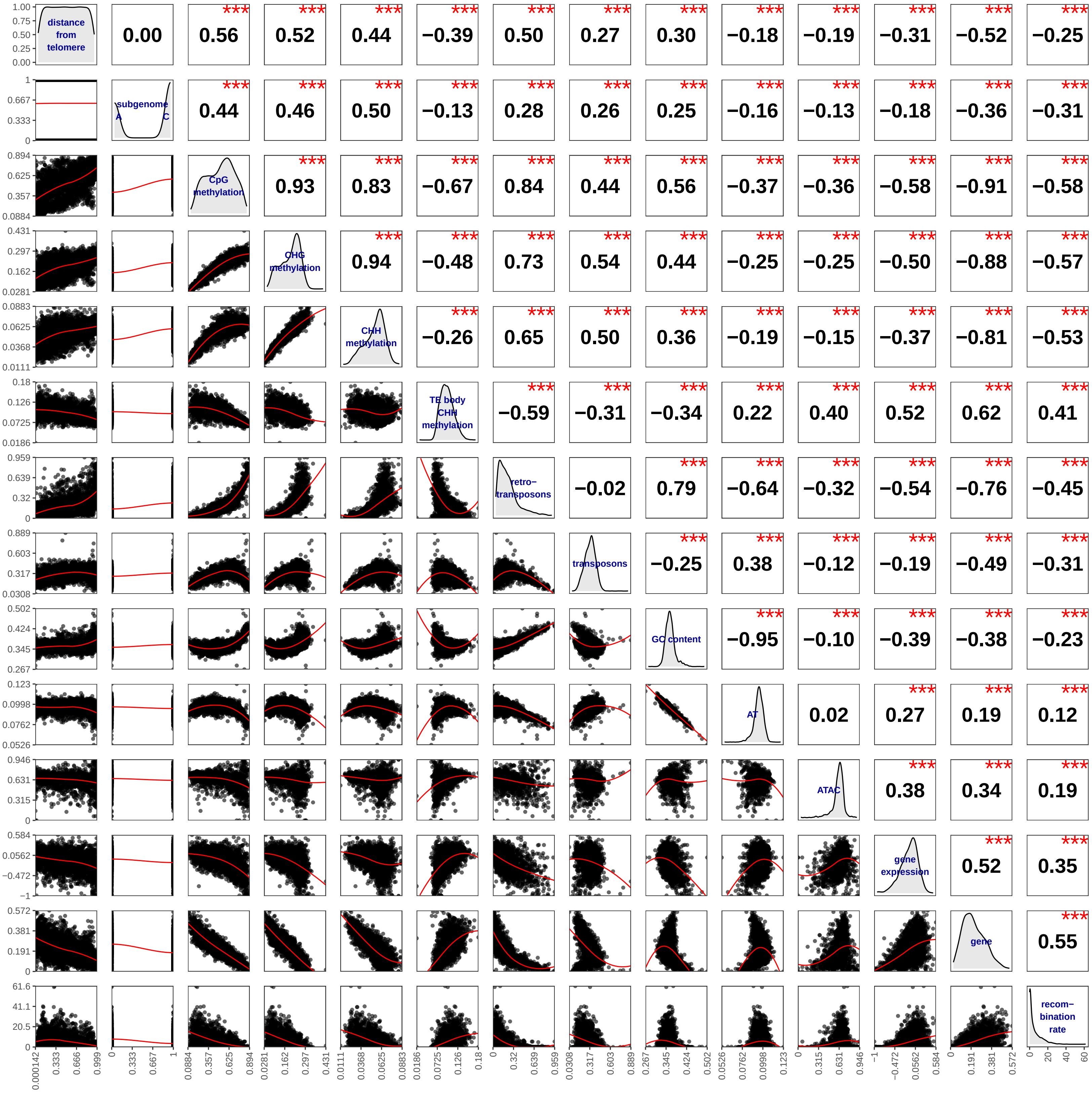
distance from telomere
0.00
0.56
0.52
0.44
−0.39
0.50
0.27
0.30
−0.18
−0.19
−0.31
−0.52
−0.25
subgenome
A
C
0.44
0.46
0.50
−0.13
0.28
0.26
0.25
−0.16
−0.13
−0.18
−0.36
−0.31
CpG methylation
0.93
0.83
−0.67
0.84
0.44
0.56
−0.37
−0.36
−0.58
−0.91
−0.58
CHG methylation
0.94
−0.48
0.73
0.54
0.44
−0.25
−0.25
−0.50
−0.88
−0.57
CHH methylation
−0.26
0.65
0.50
0.36
−0.19
−0.15
−0.37
−0.81
−0.53
TE body CHH methylation
−0.59
−0.31
−0.34
0.22
0.40
0.52
0.62
0.41
retro−transposons
−0.02
0.79
−0.64
−0.32
−0.54
−0.76
−0.45
transposons
−0.25
0.38
−0.12
−0.19
−0.49
−0.31
GC content
−0.95
−0.10
−0.39
−0.38
−0.23
AT
0.02
0.27
0.19
0.12
ATAC
0.38
0.34
0.19
gene expression
0.52
0.35
gene
0.55
recom−bination rate
***

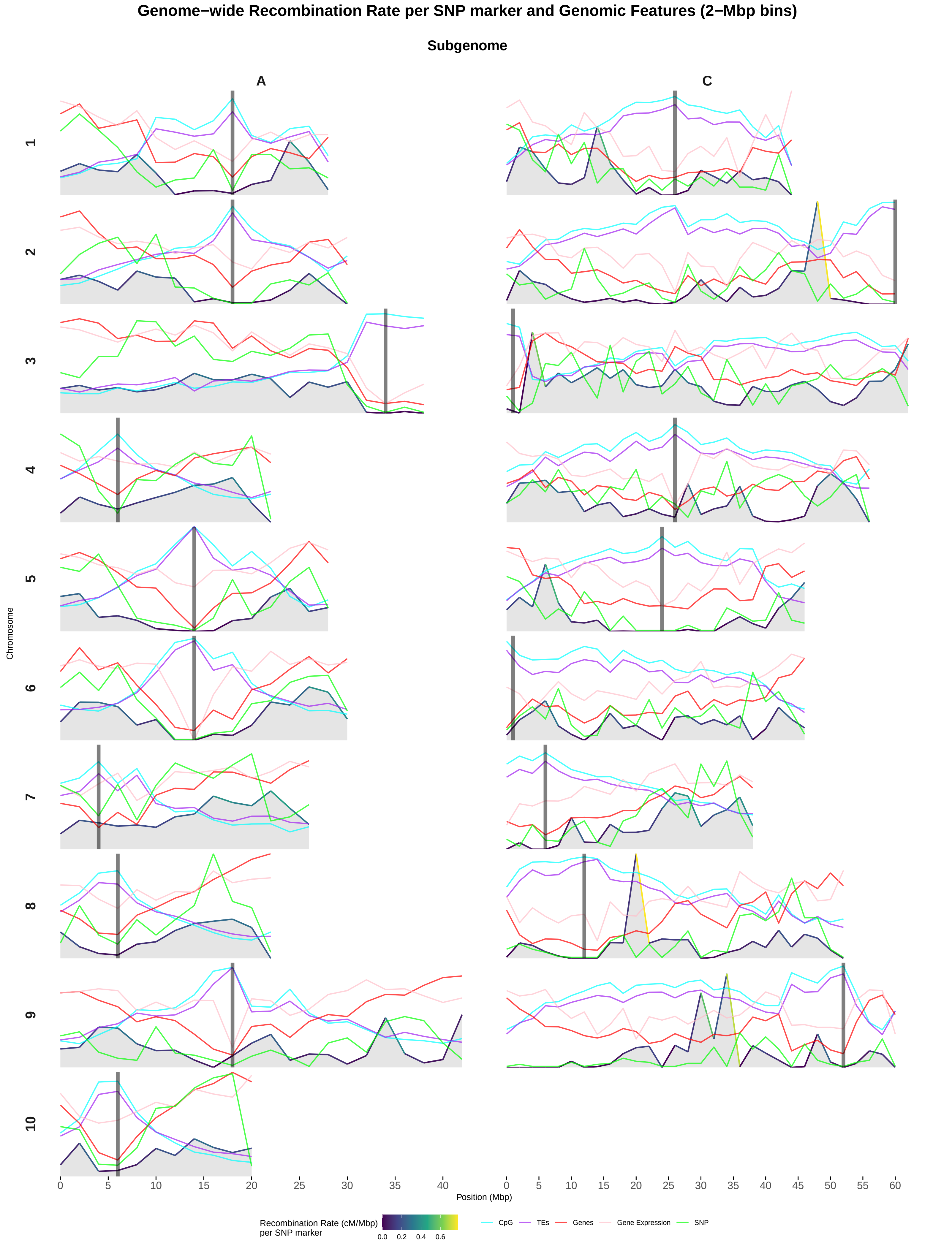

Genome−wide Recombination Rate per SNP marker and Genomic Features (2−Mbp bins)
Subgenome
A
C
Chromosome
1
2
3
4
5
6
7
8
9
10
0
5
10
15
20
25
30
35
40
45
50
55
60
Position (Mbp)
Recombination Rate (cM/Mbp)
per SNP marker
0.0
0.2
0.4
0.6
CpG
TEs
Genes
Gene Expression
SNP

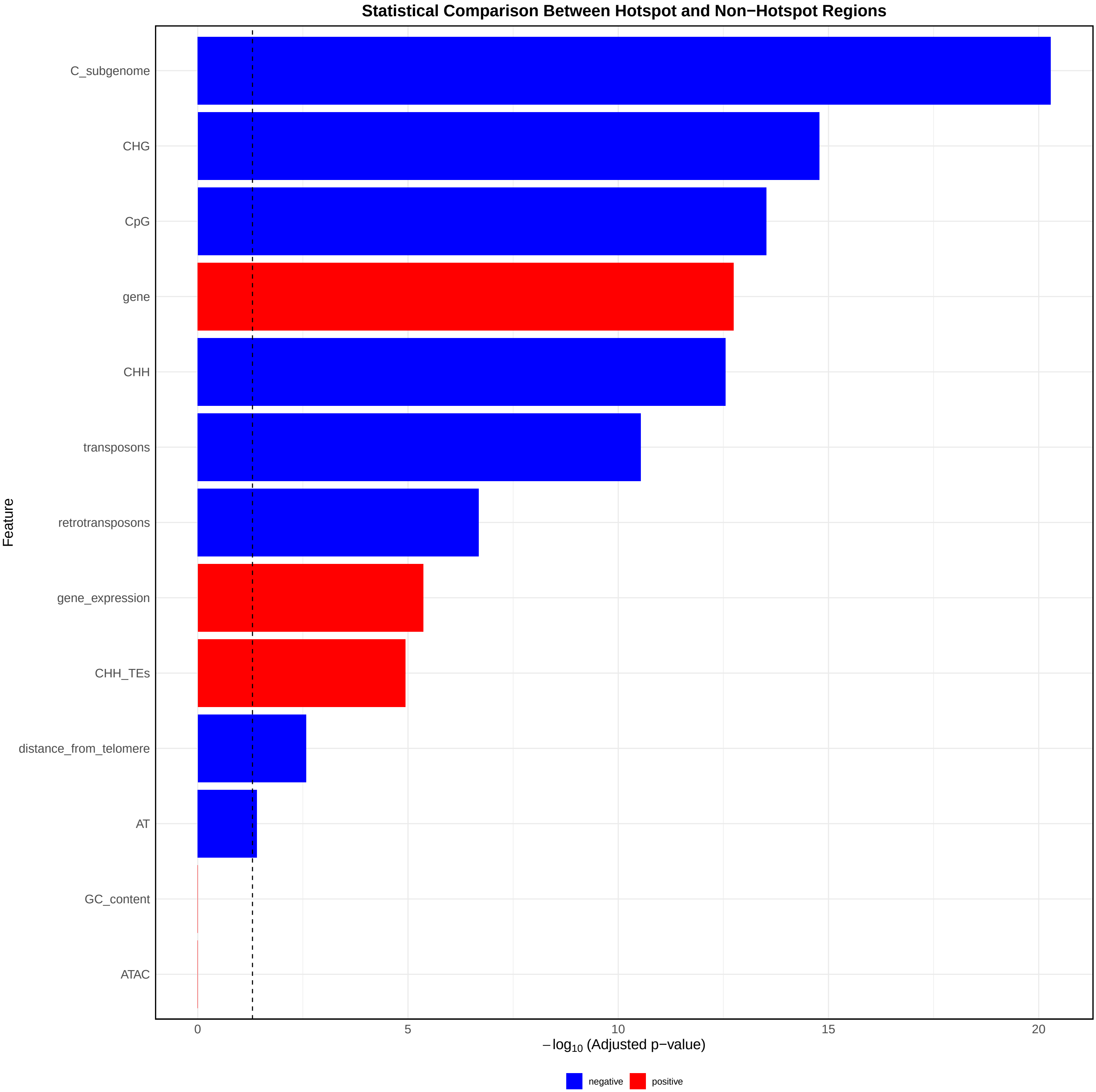

Statistical Comparison Between Hotspot and Non−Hotspot Regions
Feature
C_subgenome
CHG
CpG
gene
CHH
transposons
retrotransposons
gene_expression
CHH_TEs
distance_from_telomere
AT
GC_content
ATAC
0
5
10
15
20
−log10 (Adjusted p−value)
negative
positive

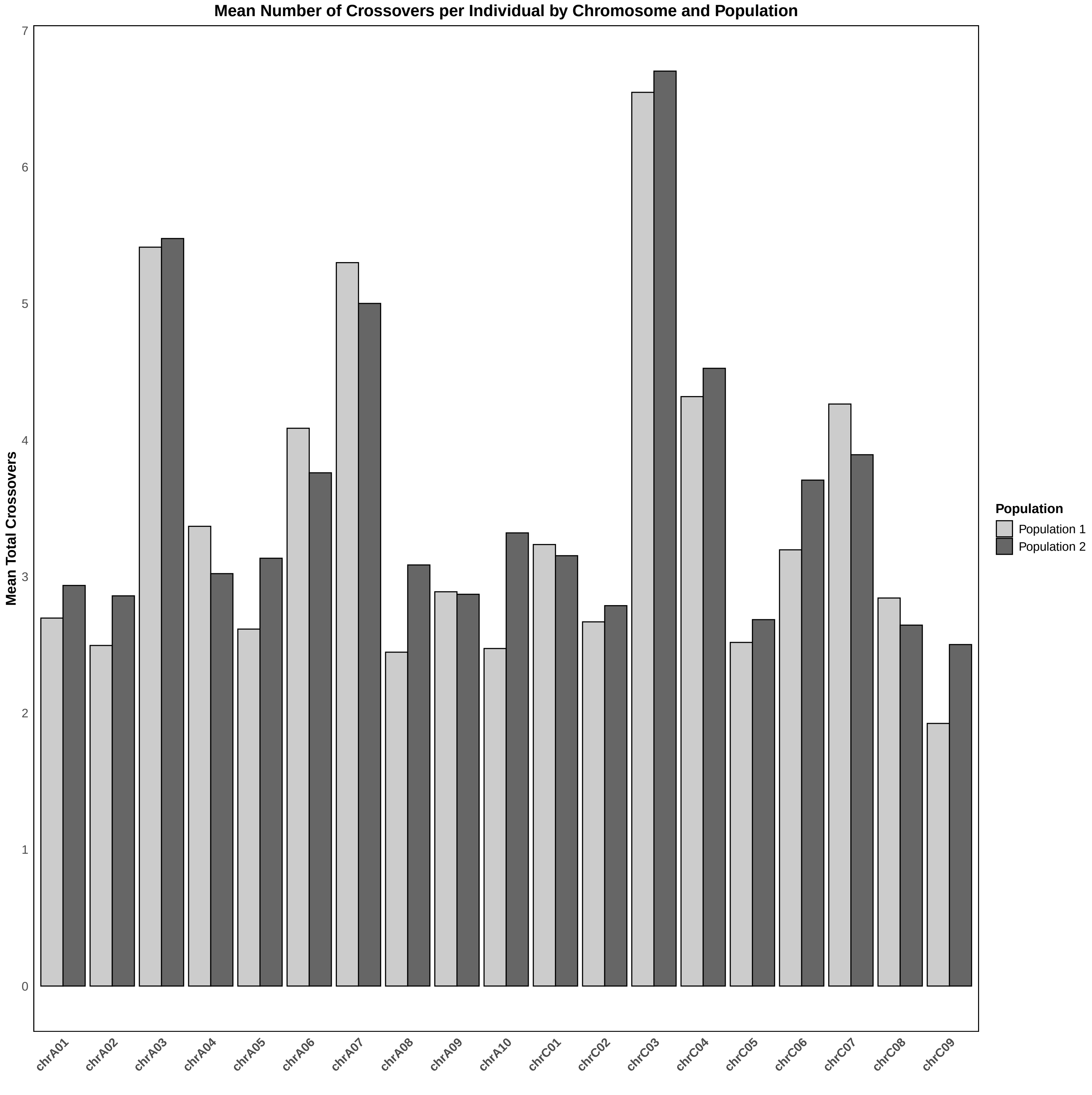

Mean Number of Crossovers per Individual by Chromosome and Population
Mean Total Crossovers
0
1
2
3
4
5
6
7
chrA01
chrA02
chrA03
chrA04
chrA05
chrA06
chrA07
chrA08
chrA09
chrA10
chrC01
chrC02
chrC03
chrC04
chrC05
chrC06
chrC07
chrC08
chrC09
Population
Population 1
Population 2

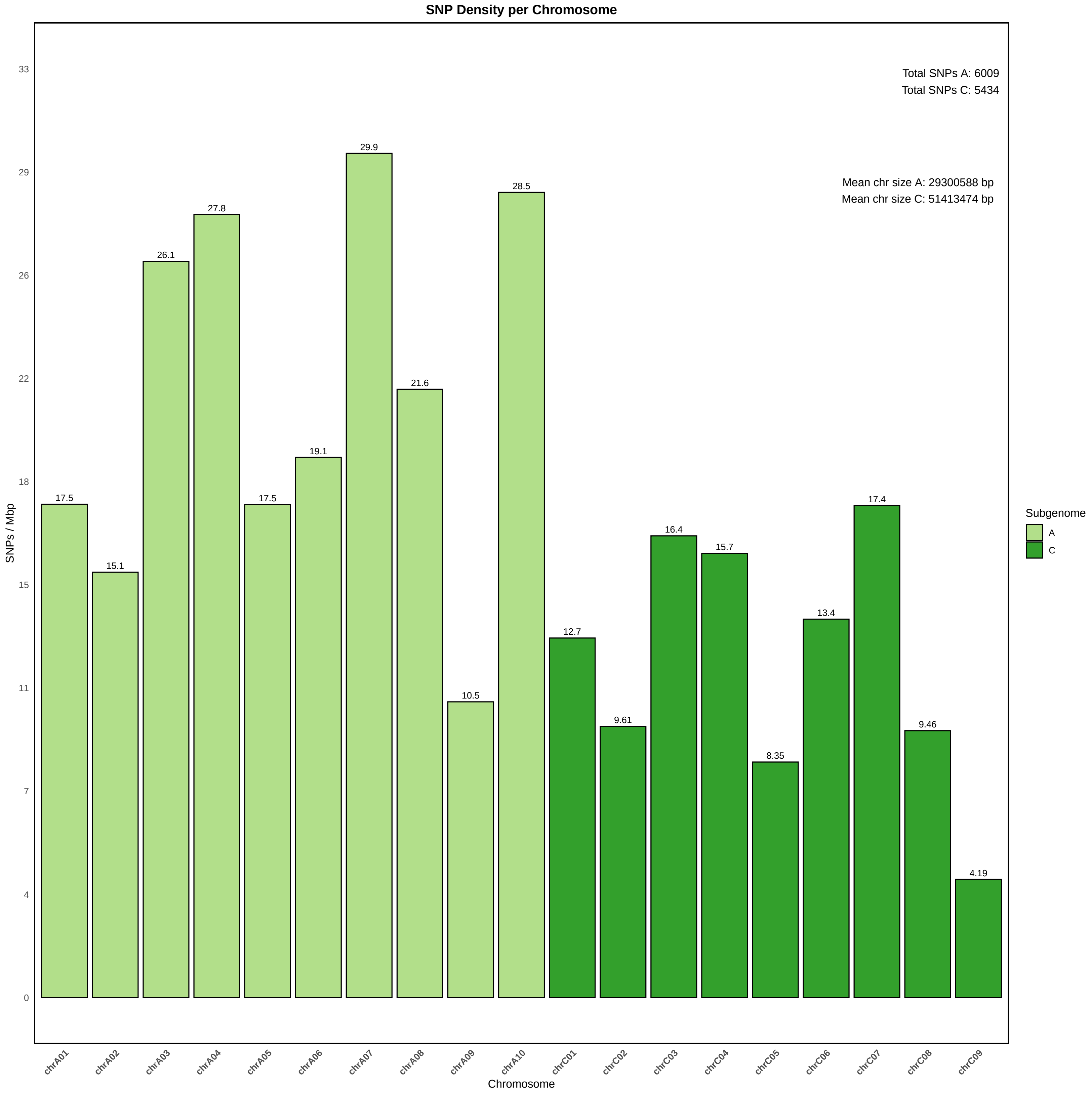
SNP Density per Chromosome
Total SNPs A: 6009
Total SNPs C: 5434
Mean chr size A: 29300588 bp
Mean chr size C: 51413474 bp
SNPs / Mbp
Chromosome
Subgenome
A
C
0
4
7
11
15
18
22
26
29
33
17.5
15.1
26.1
27.8
17.5
19.1
29.9
21.6
10.5
28.5
12.7
9.61
16.4
15.7
8.35
13.4
17.4
9.46
4.19
chrA01
chrA02
chrA03
chrA04
chrA05
chrA06
chrA07
chrA08
chrA09
chrA10
chrC01
chrC02
chrC03
chrC04
chrC05
chrC06
chrC07
chrC08
chrC09

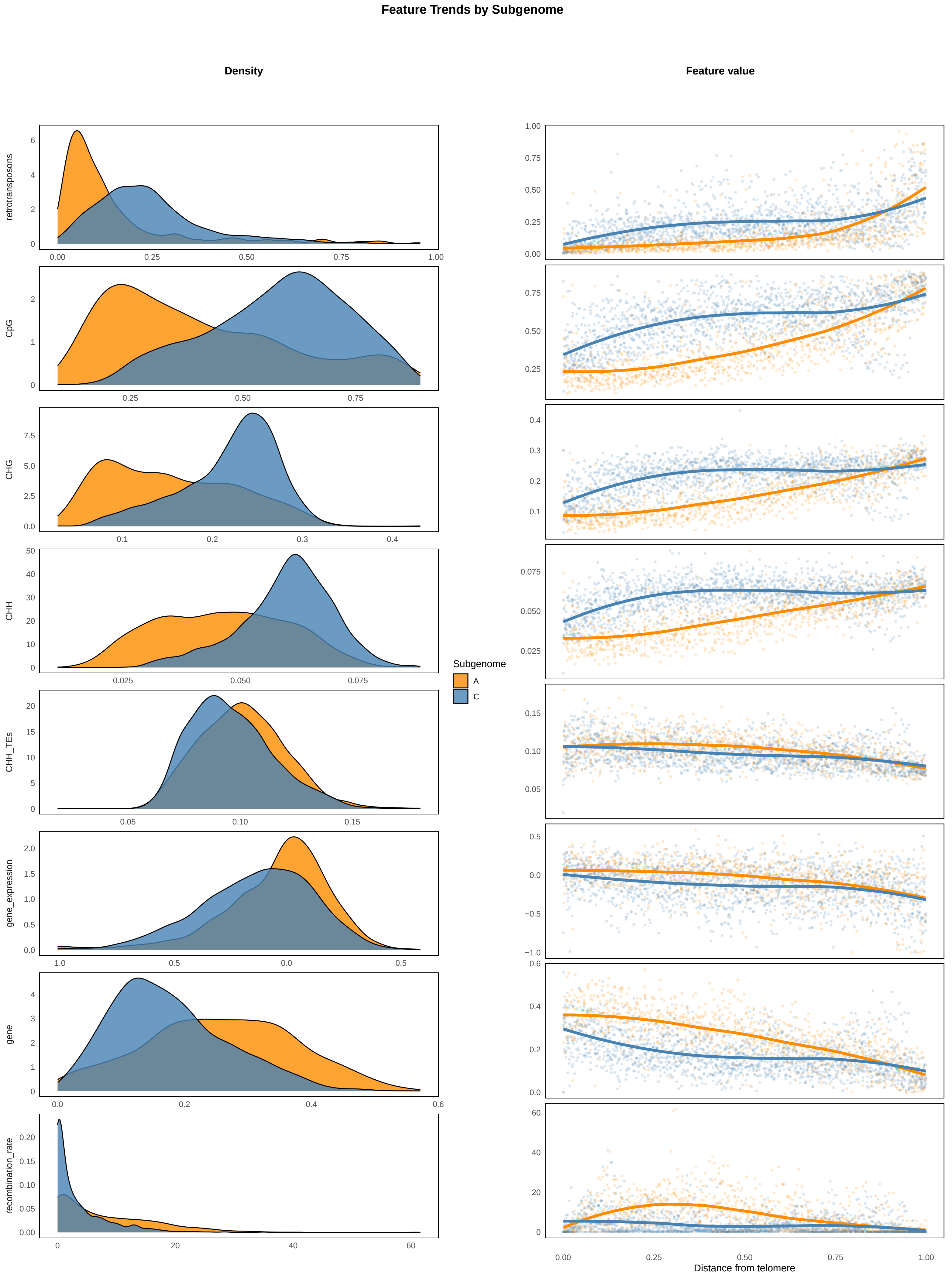

Feature Trends by Subgenome
Density
Feature value
retrotransposons
CpG
CHG
CHH
CHH_TEs
gene_expression
gene
recombination_rate
Subgenome
A
C
Distance from telomere

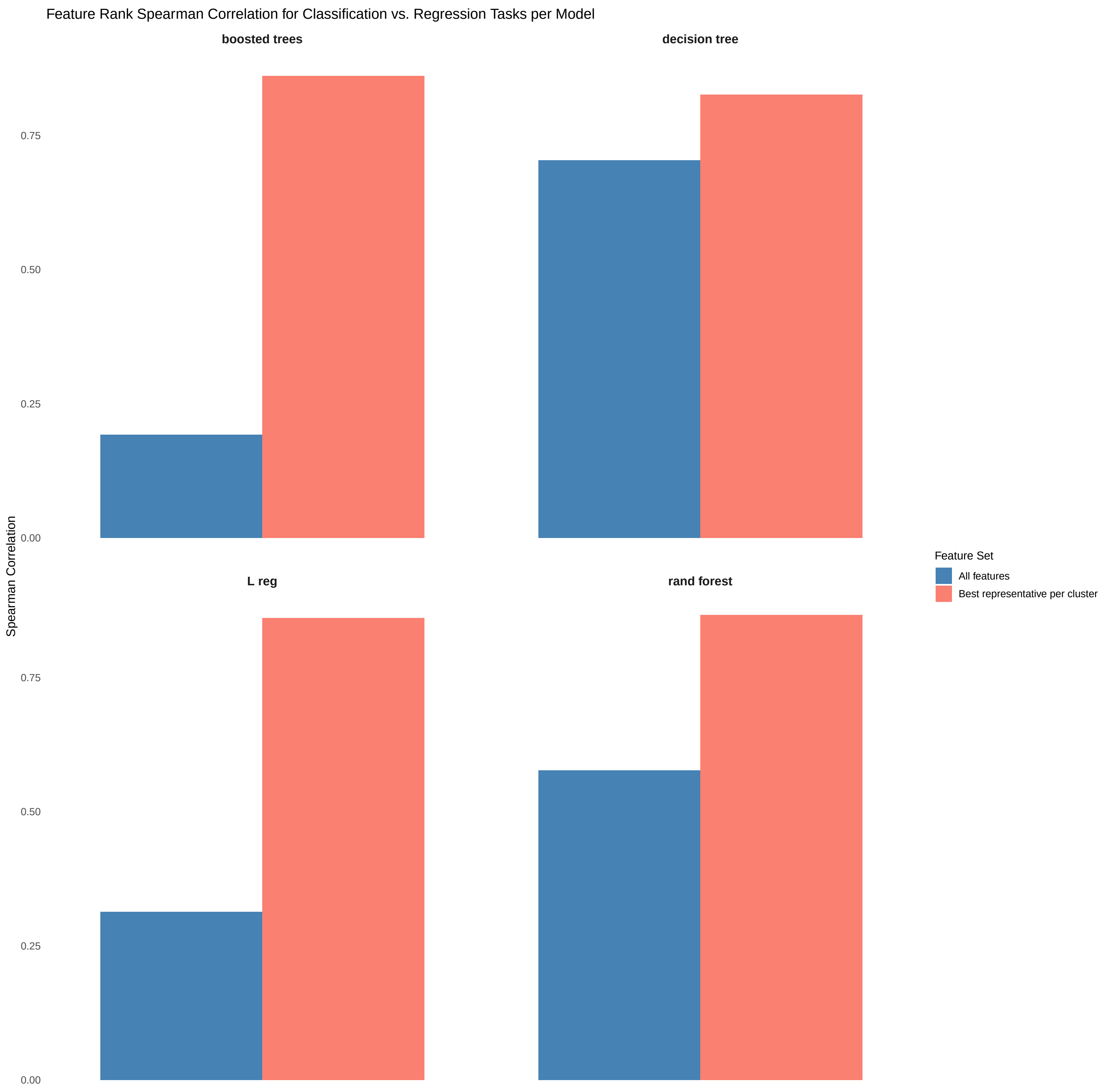
Feature Rank Spearman Correlation for Classification vs. Regression Tasks per Model
boosted trees
decision tree
L reg
rand forest
Spearman Correlation
0.00
0.25
0.50
0.75
Feature Set
All features
Best representative per cluster

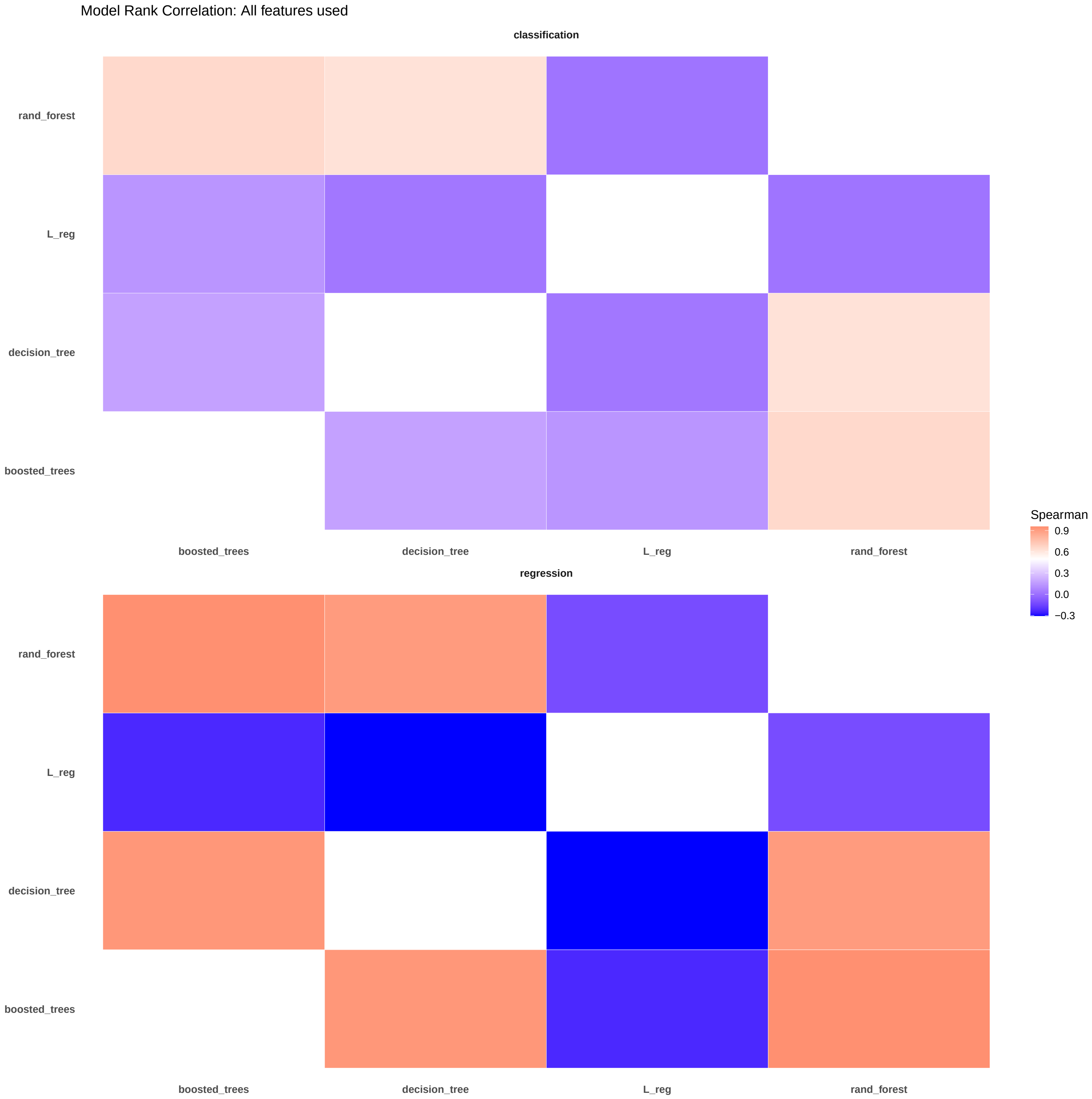
Model Rank Correlation: All features used
classification
rand_forest
L_reg
decision_tree
boosted_trees
boosted_trees
decision_tree
L_reg
rand_forest
regression
rand_forest
L_reg
decision_tree
boosted_trees
boosted_trees
decision_tree
L_reg
rand_forest
Spearman
0.9
0.6
0.3
0.0
−0.3

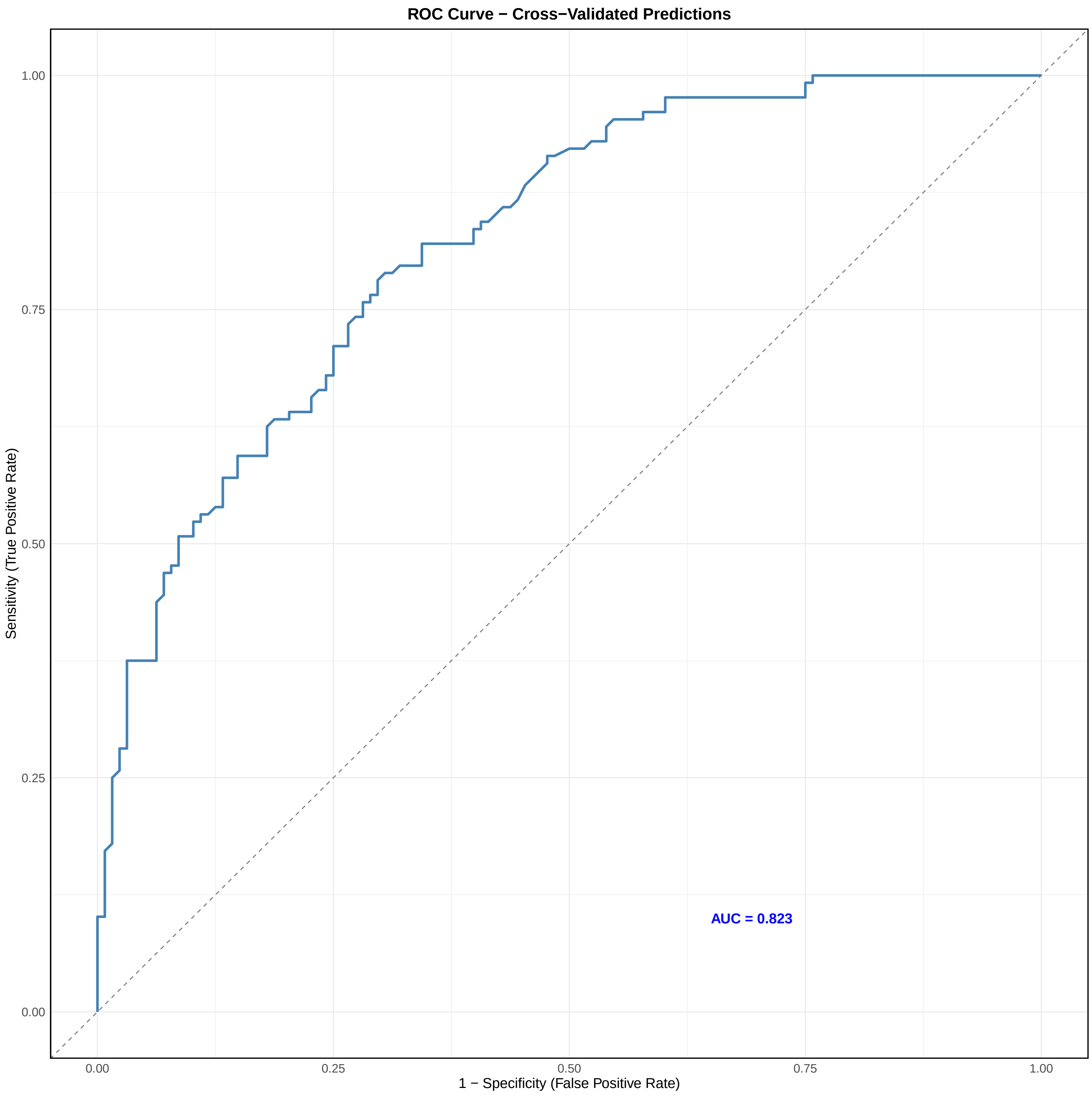

ROC Curve − Cross−Validated Predictions
1.00
0.75
0.50
0.25
0.00
Sensitivity (True Positive Rate)
0.00
0.25
0.50
0.75
1.00
1 − Specificity (False Positive Rate)
AUC = 0.823

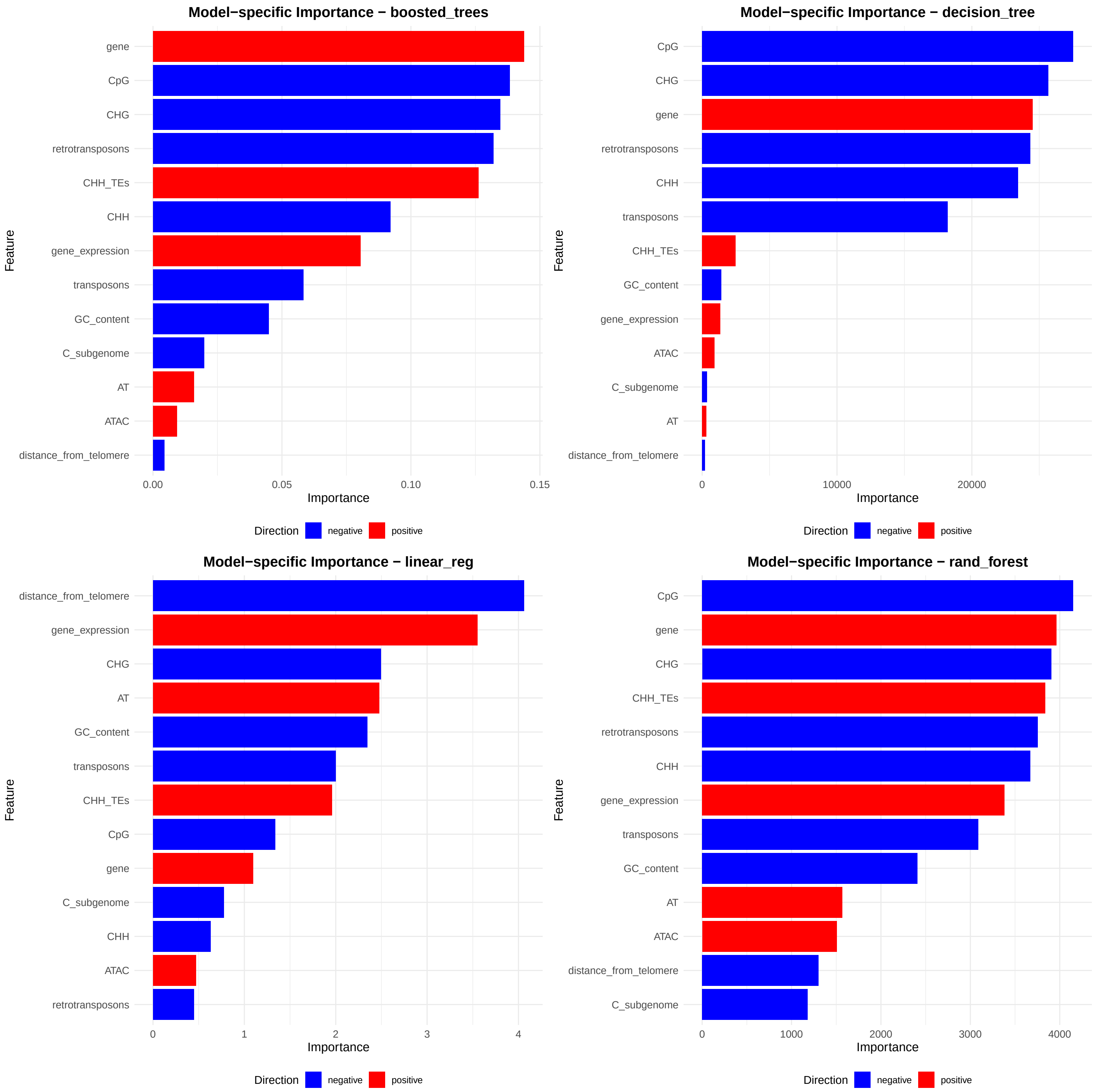
Model−specific Importance − boosted_trees
gene
CpG
CHG
retrotransposons
CHH_TEs
CHH
gene_expression
transposons
GC_content
C_subgenome
AT
ATAC
distance_from_telomere
Feature
0.00
0.05
0.10
0.15
Importance
Direction
negative
positive
Model−specific Importance − decision_tree
CpG
CHG
gene
retrotransposons
CHH
transposons
CHH_TEs
GC_content
gene_expression
ATAC
C_subgenome
AT
distance_from_telomere
Feature
0
10000
20000
Importance
Direction
negative
positive
Model−specific Importance − linear_reg
distance_from_telomere
gene_expression
CHG
AT
GC_content
transposons
CHH_TEs
CpG
gene
C_subgenome
CHH
ATAC
retrotransposons
Feature
0
1
2
3
4
Importance
Direction
negative
positive
Model−specific Importance − rand_forest
CpG
gene
CHG
CHH_TEs
retrotransposons
CHH
gene_expression
transposons
GC_content
AT
ATAC
distance_from_telomere
C_subgenome
Feature
0
1000
2000
3000
4000
Importance
Direction
negative
positive

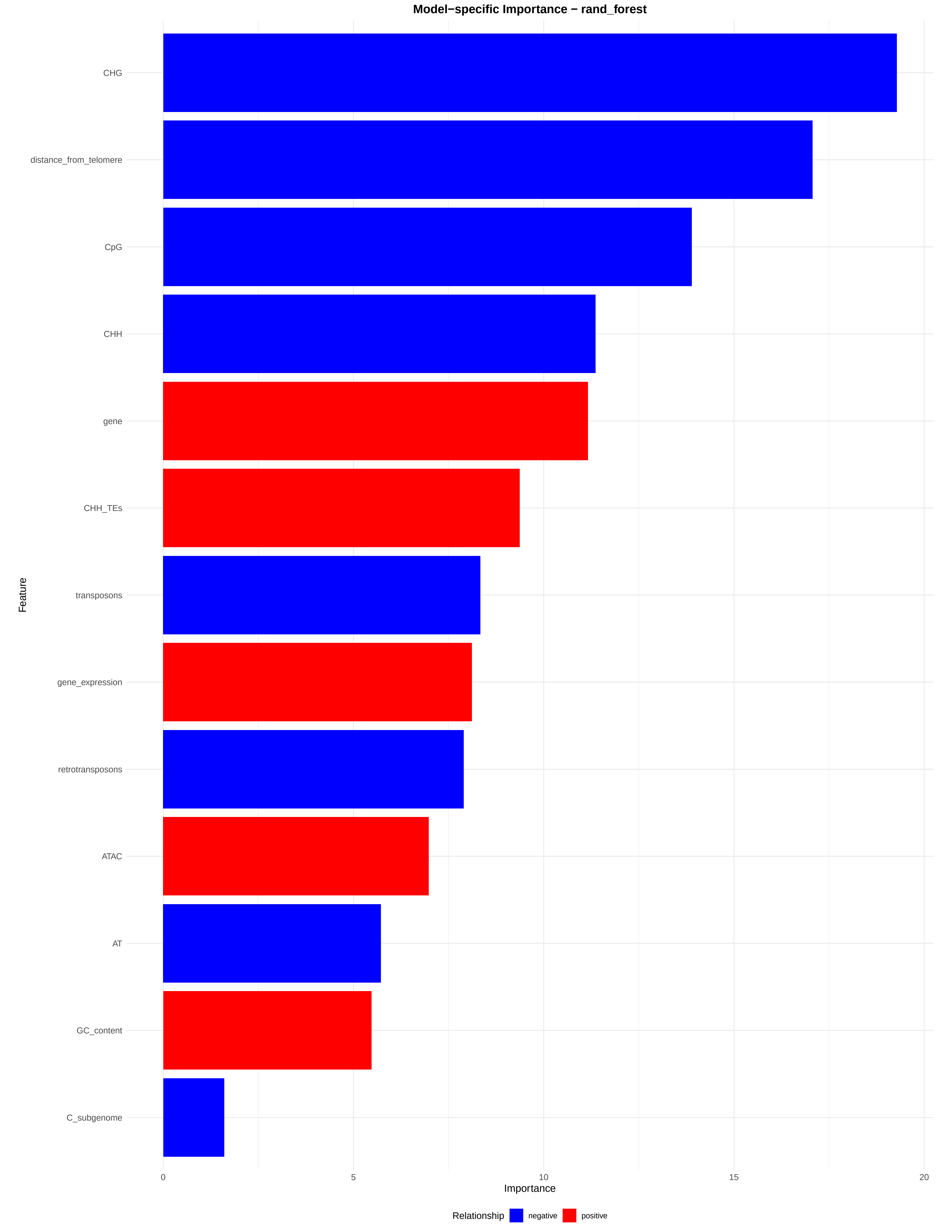

Model−specific Importance − rand_forest
CHG
distance_from_telomere
CpG
CHH
gene
CHH_TEs
transposons
gene_expression
retrotransposons
ATAC
AT
GC_content
C_subgenome
Feature
0
5
10
15
20
Importance
Relationship
negative
positive

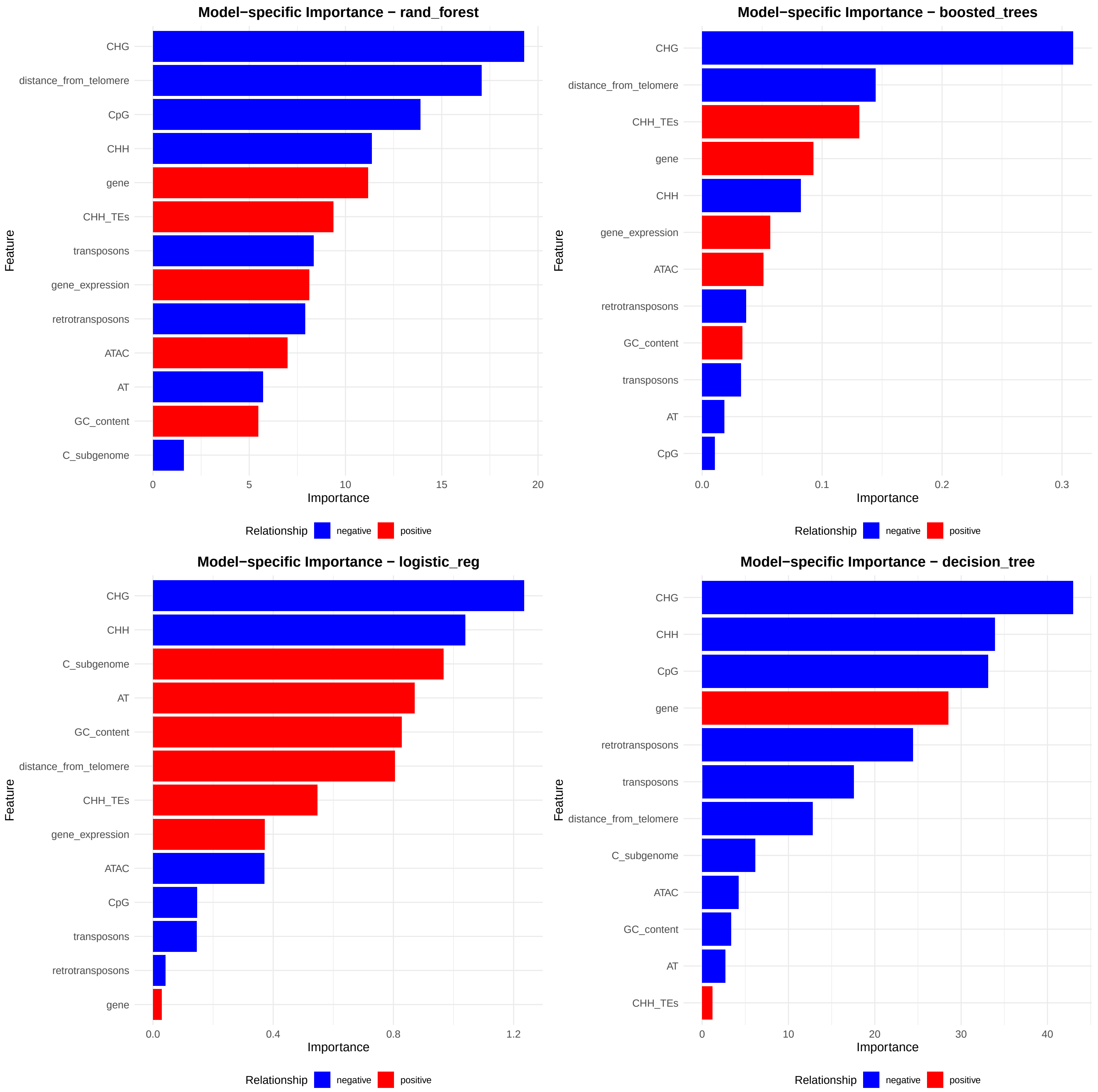

Model−specific Importance − rand_forest
CHG
distance_from_telomere
CpG
CHH
gene
CHH_TEs
transposons
gene_expression
retrotransposons
ATAC
AT
GC_content
C_subgenome
Feature
0
5
10
15
20
Importance
Relationship
negative
positive
Model−specific Importance − boosted_trees
CHG
distance_from_telomere
CHH_TEs
gene
CHH
gene_expression
ATAC
retrotransposons
GC_content
transposons
AT
CpG
Feature
0.0
0.1
0.2
0.3
Importance
Relationship
negative
positive
Model−specific Importance − logistic_reg
CHG
CHH
C_subgenome
AT
GC_content
distance_from_telomere
CHH_TEs
gene_expression
ATAC
CpG
transposons
retrotransposons
gene
Feature
0.0
0.4
0.8
1.2
Importance
Relationship
negative
positive
Model−specific Importance − decision_tree
CHG
CHH
CpG
gene
retrotransposons
transposons
distance_from_telomere
C_subgenome
ATAC
GC_content
AT
CHH_TEs
Feature
0
10
20
30
40
Importance
Relationship
negative
positive

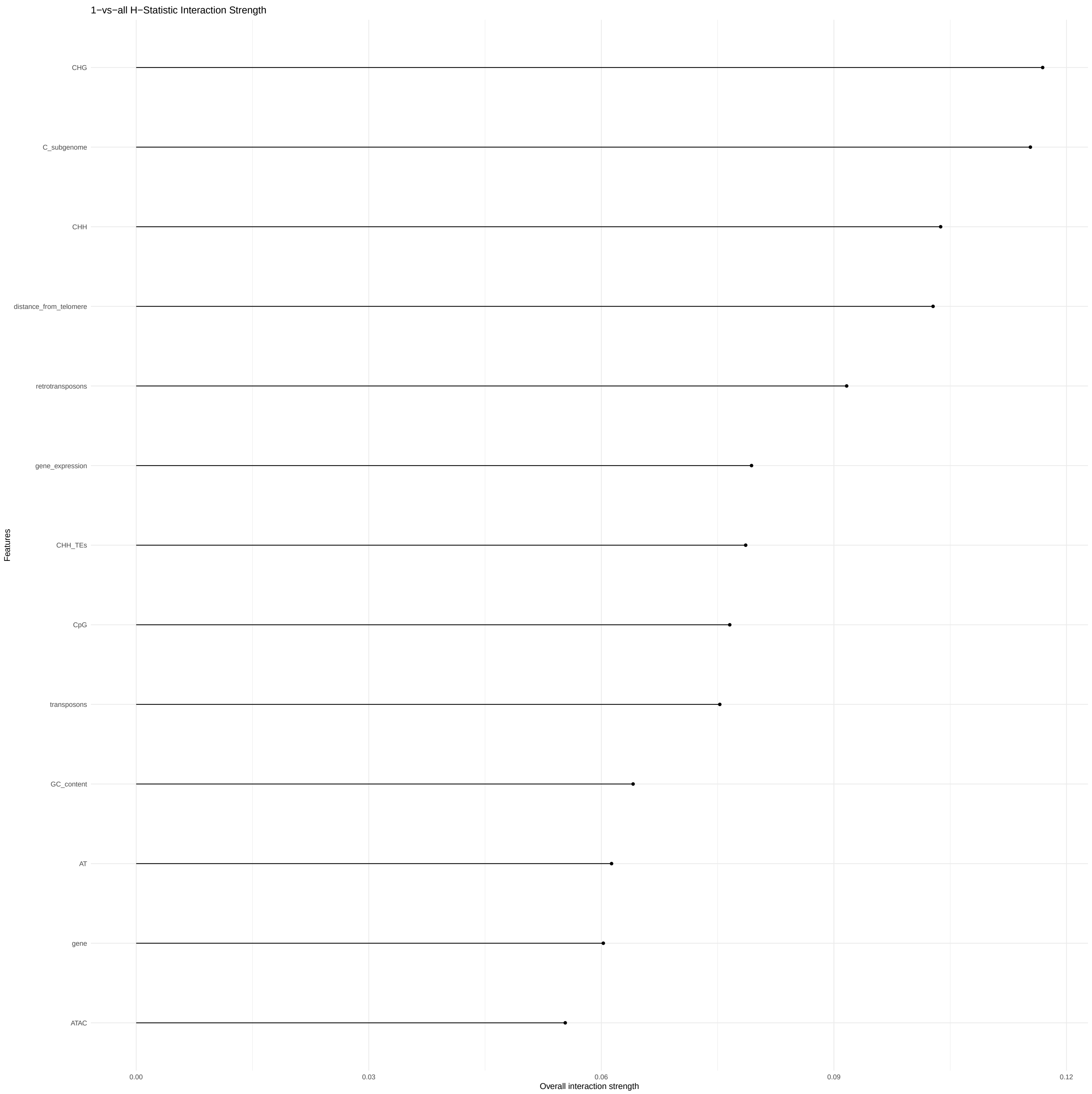
1−vs−all H−Statistic Interaction Strength
CHG
C_subgenome
CHH
distance_from_telomere
retrotransposons
gene_expression
CHH_TEs
CpG
transposons
GC_content
AT
gene
ATAC
Features
0.00
0.03
0.06
0.09
0.12
Overall interaction strength

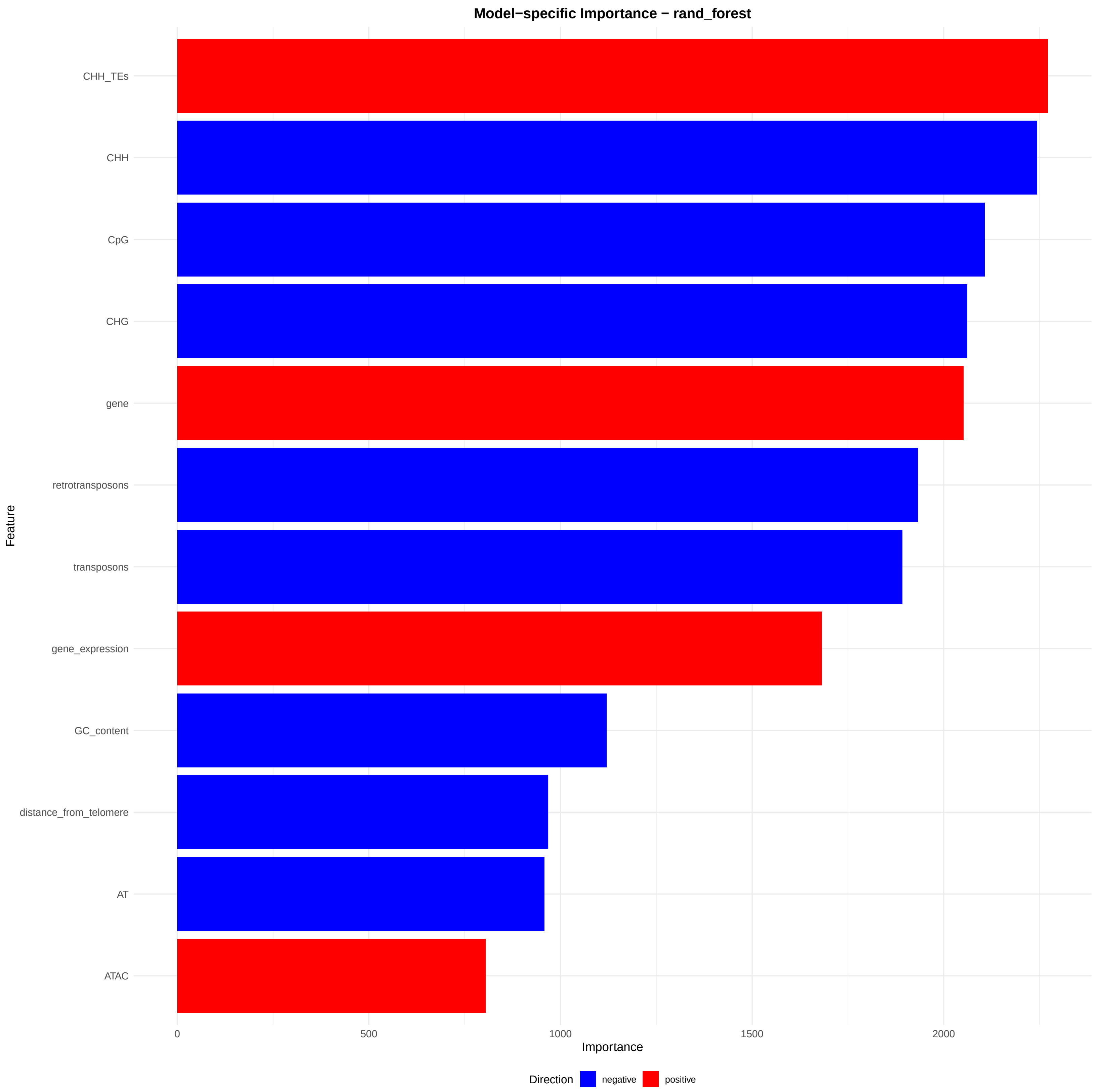
Model−specific Importance − rand_forest
CHH_TEs
CHH
CpG
CHG
gene
retrotransposons
transposons
gene_expression
GC_content
distance_from_telomere
AT
ATAC
Feature
0
500
1000
1500
2000
Importance
Direction
negative
positive

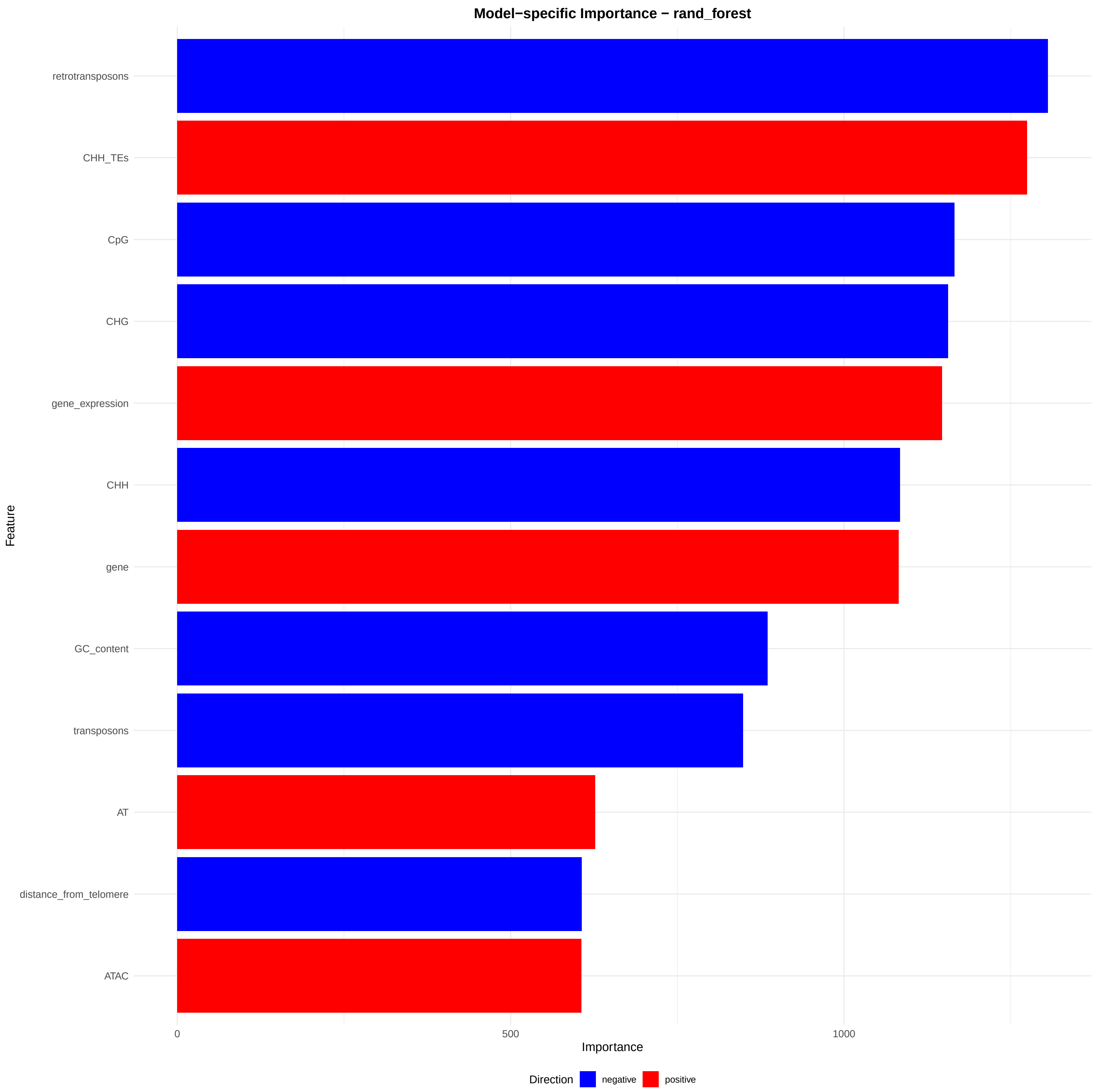

Model−specific Importance − rand_forest
retrotransposons
CHH_TEs
CpG
CHG
gene_expression
CHH
gene
GC_content
transposons
AT
distance_from_telomere
ATAC
Feature
0
500
1000
Importance
Direction
negative
positive

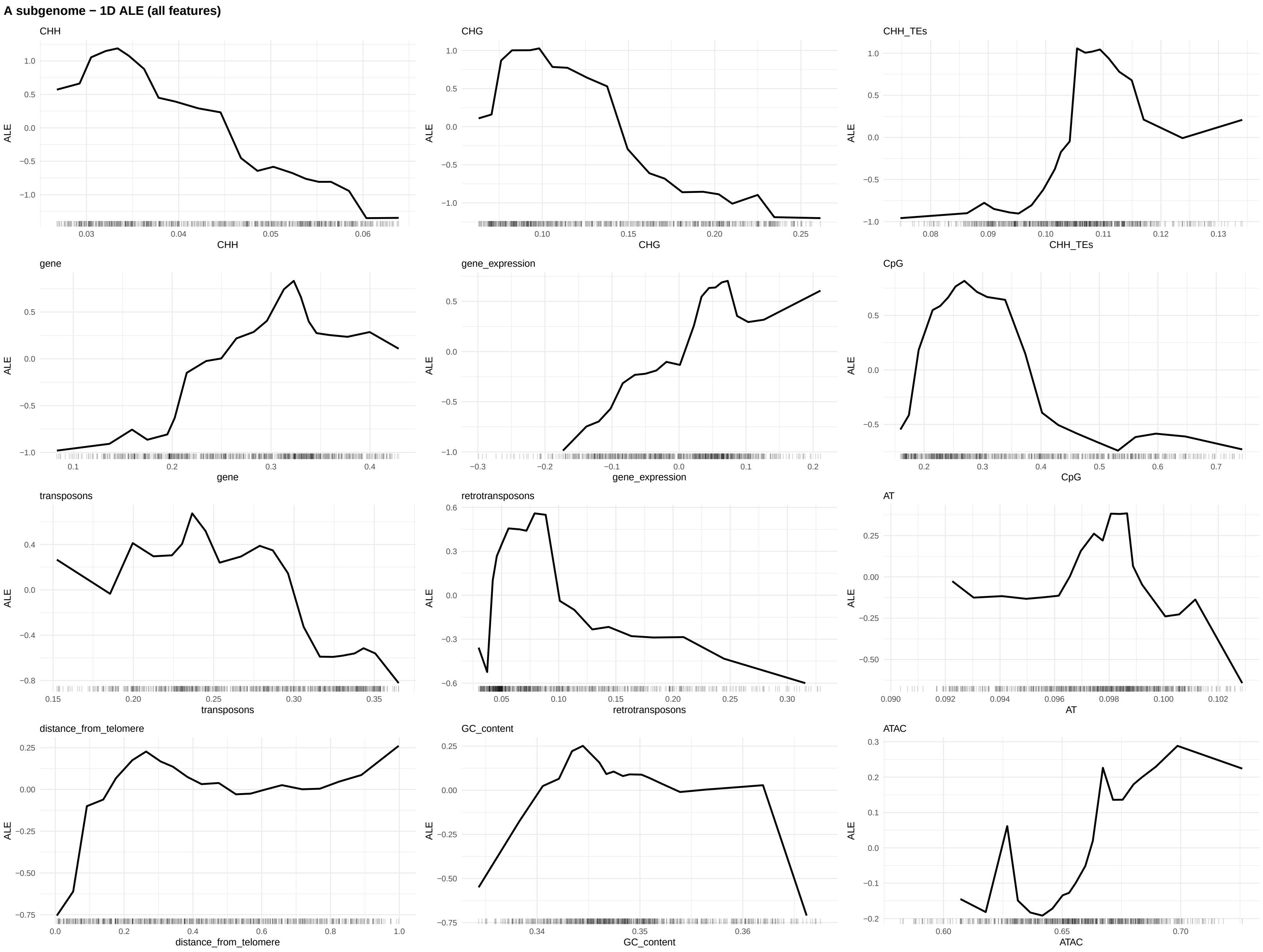

A subgenome − 1D ALE (all features)
CHH
CHG
CHH_TEs
gene
gene_expression
CpG
transposons
retrotransposons
AT
distance_from_telomere
GC_content
ATAC
ALE

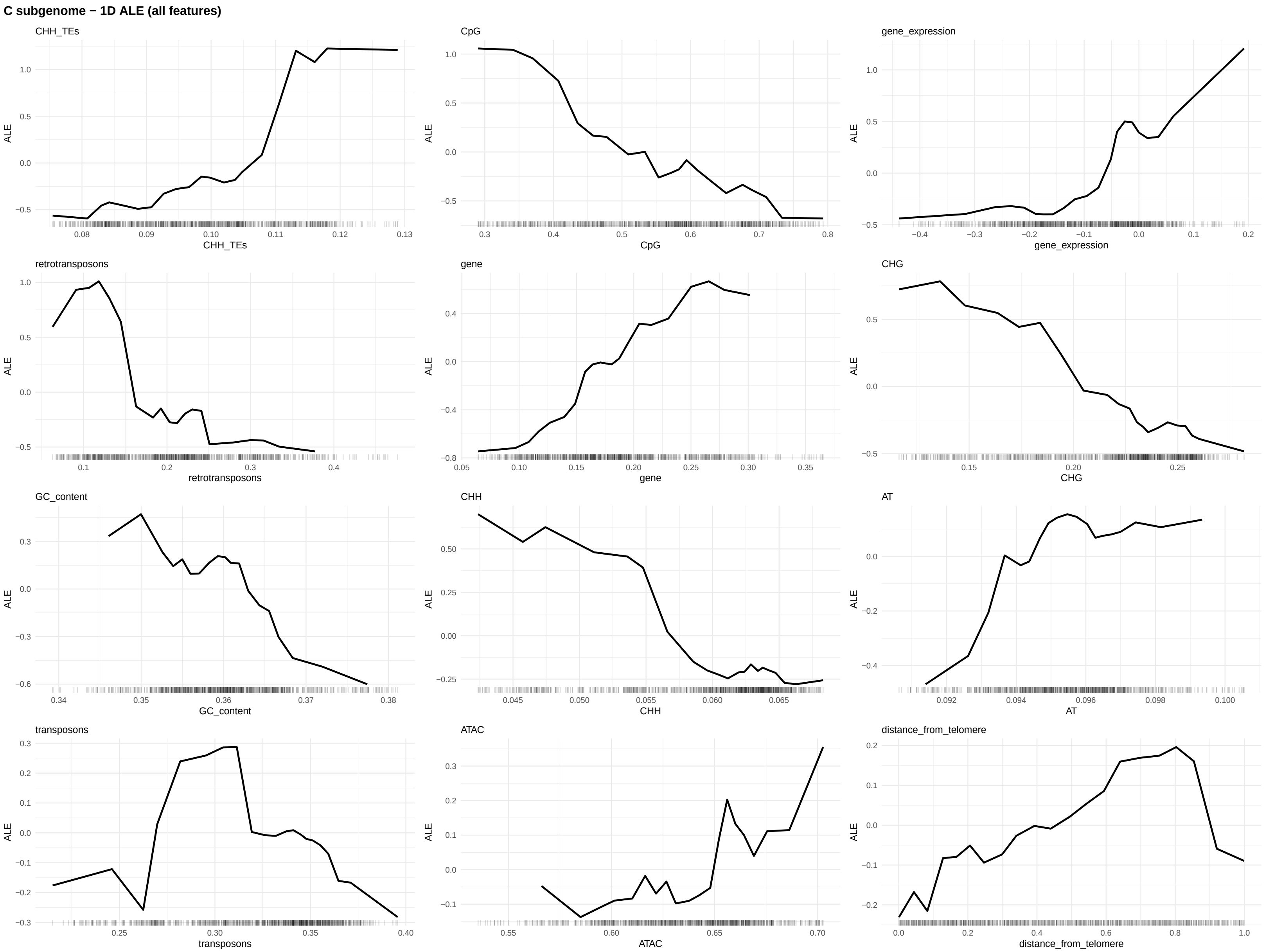

C subgenome − 1D ALE (all features)
CHH_TEs
CpG
gene_expression
retrotransposons
gene
CHG
GC_content
CHH
AT
transposons
ATAC
distance_from_telomere
ALE

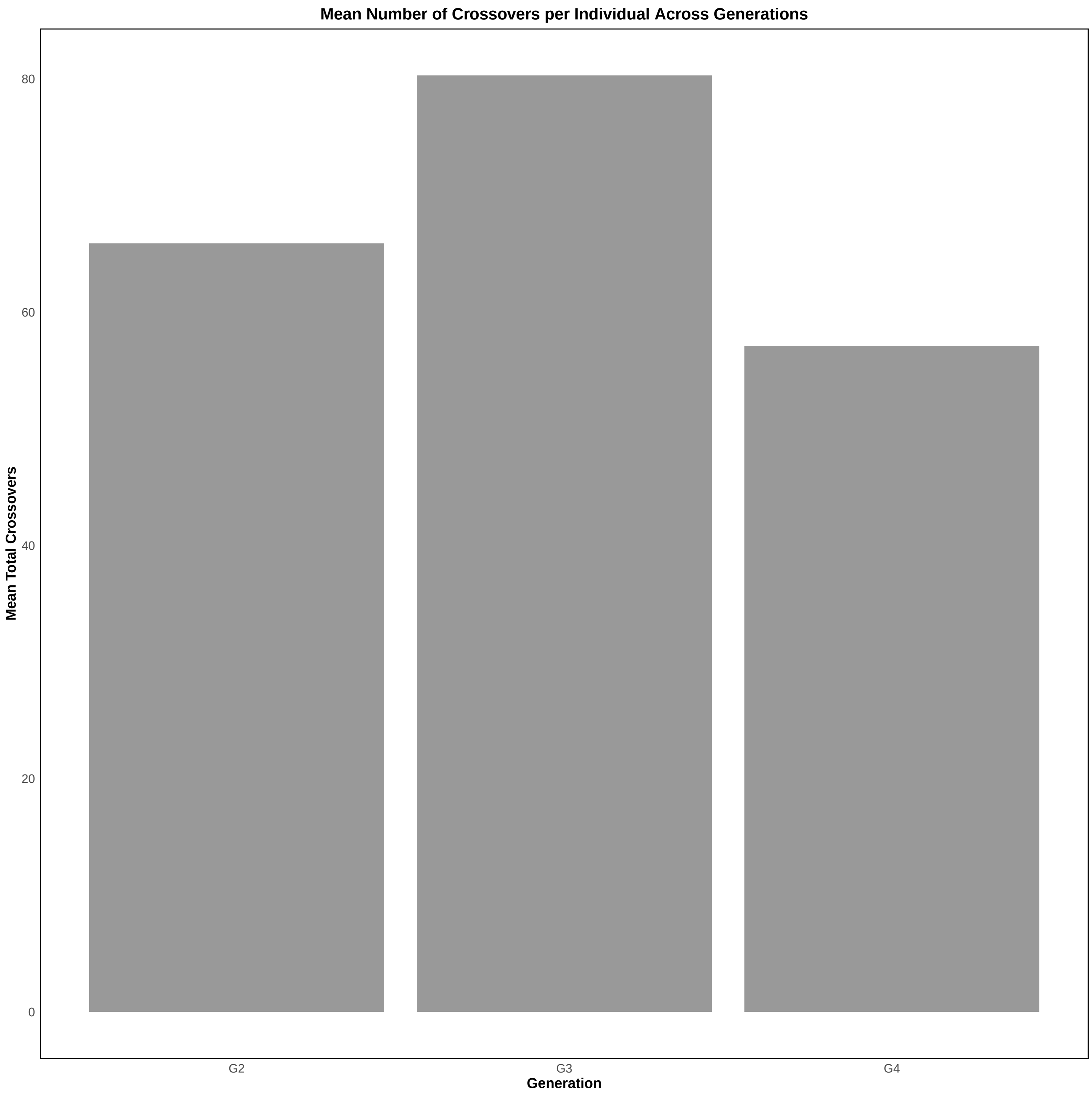
Mean Number of Crossovers per Individual Across Generations
Mean Total Crossovers
80
60
40
20
0
G2
G3
G4
Generation

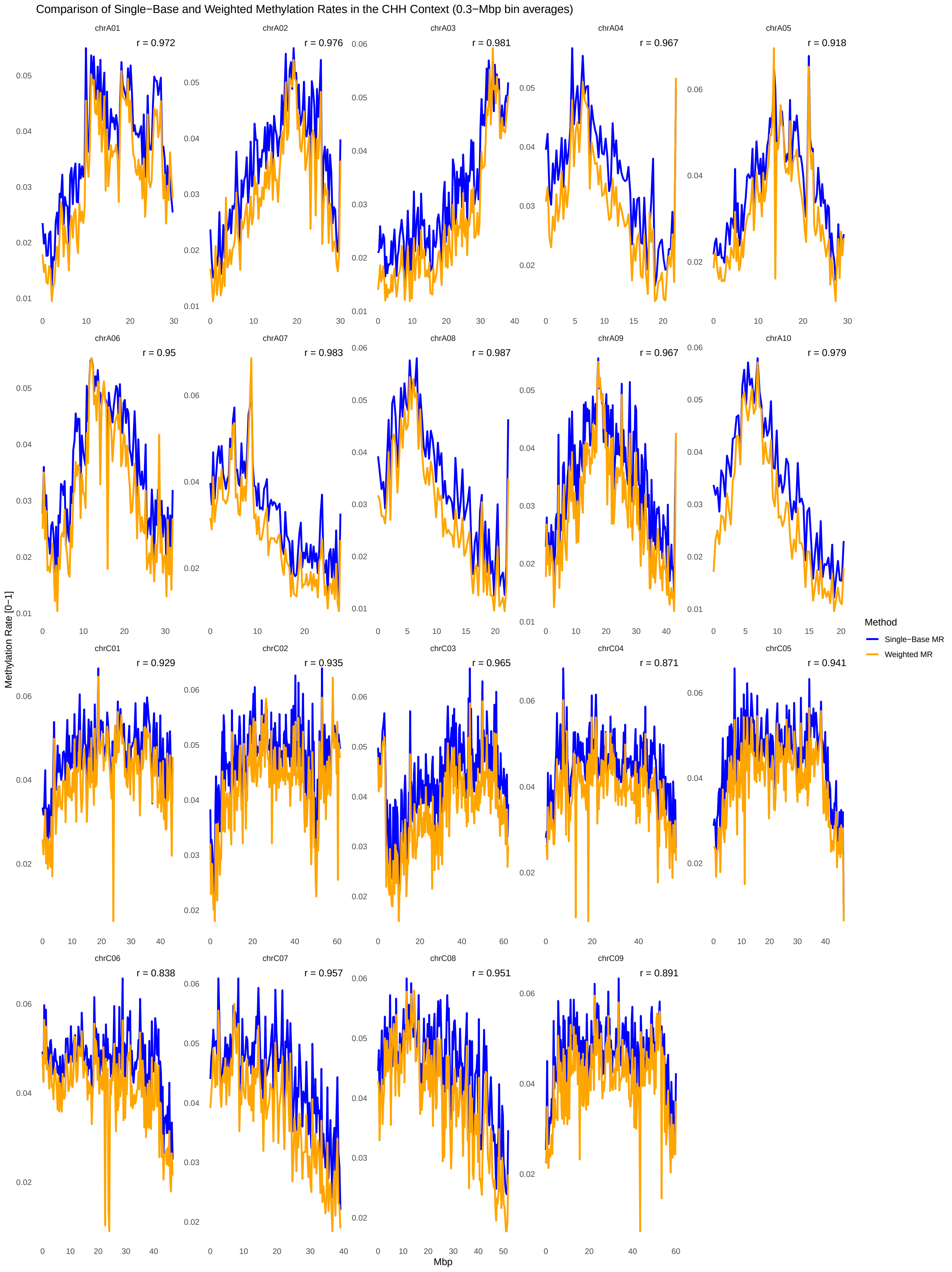

Comparison of Single−Base and Weighted Methylation Rates in the CHH Context (0.3−Mbp bin averages)
chrA01
r = 0.972
chrA02
r = 0.976
chrA03
r = 0.981
chrA04
r = 0.967
chrA05
r = 0.918
chrA06
r = 0.95
chrA07
r = 0.983
chrA08
r = 0.987
chrA09
r = 0.967
chrA10
r = 0.979
chrC01
r = 0.929
chrC02
r = 0.935
chrC03
r = 0.965
chrC04
r = 0.871
chrC05
r = 0.941
chrC06
r = 0.838
chrC07
r = 0.957
chrC08
r = 0.951
chrC09
r = 0.891
Methylation Rate [0−1]
Mbp
Method
Single−Base MR
Weighted MR
